# ^Me^CAAC=N^−^: A Cyclic (Alkyl)(Amino)Carbene Imino Ligand

**DOI:** 10.1002/chem.201904715

**Published:** 2020-01-09

**Authors:** James T. Goettel, Haopeng Gao, Simon Dotzauer, Holger Braunschweig

**Affiliations:** ^1^ Institute for Inorganic Chemistry Julius-Maximilians-Universität Würzburg Am Hubland 97074 Würzburg Germany; ^2^ Institute for Sustainable Chemistry & Catalysis with Boron Julius-Maximilians-Universität Würzburg Am Hubland 97074 Würzburg Germany

**Keywords:** boron, carbenes, imide ligands, nitrogen ligands, titanium

## Abstract

A cyclic (alkyl)(amino)carbene (CAAC) has been shown to react with a covalent azide similar to the Staudinger reaction. The reaction of ^Me^CAAC with trimethylsilyl azide afforded the N‐silylated 2‐iminopyrrolidine (^Me^CAAC=NSiMe_3_), which was fully characterized. This compound undergoes hydrolysis to afford the 2‐iminopyrrolidine and trimethylsiloxane which co‐crystallize as a hydrogen‐bonded adduct. The N‐silylated 2‐iminopyrrolidine was used to transfer the novel pyrrolidine‐2‐iminato ligand onto both main‐group and transition‐metal centers. The reaction of the tetrabromodiborane bis(dimethyl sulfide) adduct with two equivalents of ^Me^CAAC=NSiMe_3_ afforded the disubstituted diborane. The reaction of ^Me^CAAC=NSiMe_3_ with TiCl_4_ and CpTiCl_3_ afforded ^Me^CAAC=NTiCl_3_ and ^Me^CAAC=NTiCl_2_Cp, respectively.

## Graphical abstract

**Electron‐rich iminato ligands**: Reaction of a cyclic (alkyl)(amino)carbene (CAAC) with trimethylsilylazide provides a convenient reagent for transferring a CAAC=N^−^ iminato ligand to main‐group and transition‐metal fragments through facile halosilane elimination.

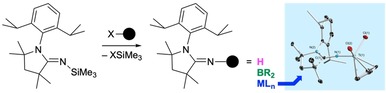

## Introduction

**Figure 1:**
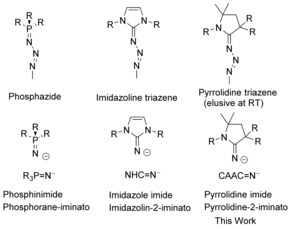
N‐Donor ligands synthesized through Staudinger‐like reactions and their triazene intermediates. R denotes a range of substituents such as alkyls and aromatics.

In 1919, Staudinger and Meyer first reported their seminal work on the reaction of triaryl phosphanes with covalent azides to form phosphinimides.^1^ Since that time, the Staudinger reaction has played important roles in organic chemistry and led to the development of the Staudinger ligation, which is used extensively in chemical biology.^2^ In certain instances, an intermediate in the Staudinger reaction, a phosphazide (Figure 1), can be isolated.^3^ Similarly, N‐heterocyclic carbenes have been shown to react with covalent azides to form stable triazenes,^4^ which could then be thermally decomposed to the imidazole imides. Phosphorane‐iminato (phosphinimide) ligands have been widely used in both transition‐metal complexes^5^ and main‐group compounds.^6^ Similar to phosphorane iminato ligands, imidazolin‐2‐iminato ligands^7^ have been used in a wide variety of transition metal complexes.^8^ These monoanionic ligands (ImN^−^) are highly basic and can act as a 2σ‐, 2π‐ or as 2σ‐, 4π‐electron donors. N‐heterocyclic olefins (NHOs) share similar strong electron donating properties and have seen a resurgence in recent years.^9^

Imidazolin‐2‐iminato ligands have recently been used in f‐block chemistry^10^ and the resulting complexes have shown remarkable catalytic activity.^11^ The ligands have also started to find use in stabilizing a variety of interesting main‐group species. For example, due to their strong π‐donor and σ‐donor properties, these ligands were able to stabilize and allow isolation of a singlet phosphinonitrene.^12^ More recently, this ligand type has been used to obtain a variety of unusual germanium,^13^ aluminum,^14^ silicon,^15^ tin,^16^ and other compounds.^17^ Several imidazolin‐2‐iminato‐supported boron compounds have been shown to act as catalysts for the metal‐free dehydrogenation of amine‐boranes.^18^ Very recently, Inoue and co‐workers also used a bulky imidazolin‐2‐iminato ligand to stabilize a borasilene,^19^ and Dielmann and co‐workers were able to isolate and characterize three‐coordinate phosphorus dications.^20^ This class of ligands has also been used in the synthesis of superbasic phosphines, which were subsequently shown to split CO_2_ and activate SF_6_.^21^

Cyclic (alkyl)(amino)carbenes (CAACs) are stronger σ‐donors and π‐acceptors than N‐heterocyclic carbenes (NHCs).^22^ They have been used to stabilize a wide range of highly reactive species and have been shown to be useful ligands for transition‐metal catalysis.^23^ Despite their extensive use in main‐group chemistry, reactions between CAACs and covalent azides have not been reported and we were unable to find examples of the pyrrolidine‐2‐iminato ligand in main‐group or transition‐metal chemistry. In light of the success of and interest in imidazolin‐2‐iminato ligands, we sought to explore the possibility of synthesizing a pyrrolidine‐2‐iminato transfer reagent and its use as a ligand in both main‐group and transition‐metal chemistry.

Herein, we report the facile room temperature reaction of ^Me^CAAC with Me_3_SiN_3_, which readily affords ^Me^CAAC=NSiMe_3_ under mild conditions. In simple procedures, the anionic pyrrolidine‐2‐iminato ligand derived therefrom was subsequently bound to the boron and titanium centers of a number of compounds, and these were characterized by NMR spectroscopy and X‐ray crystallography.

## Results and Discussion

**Scheme 1:**
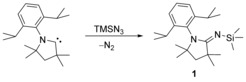
Reaction between ^Me^CAAC and TMSN_3_ to afford ^Me^CAAC=NSiMe_3_ (**1**).

The N‐silylated 2‐iminopyrrolidine ^Me^CAAC=NSiMe_3_ (**1**) was synthesized by the reaction of ^Me^CAAC with TMSN_3_ (trimethylsilyl azide) at room temperature with the loss of N_2_ (Scheme 1). Evaporation of the solvent afforded pure **1** as a highly crystalline, colorless solid in essentially quantitative yield, with no discernable side‐products. An excess of TMSN_3_ helped ensure that the reaction proceeded to completion and did not result in any byproducts. In contrast to N‐heterocyclic carbenes, which generally require high temperatures and long reaction times to convert the triazene to the imine,^8^ the imine **1** is formed rapidly with minimal heating. At room temperature, the mixture of ^Me^CAAC and TMSN_3_ in benzene develops a slight shade of yellow, which dissipates after the reaction is complete. This is corroborated by the observation of new weak signals in the ^1^H NMR spectrum of the mixture, which disappear after the reaction is complete and suggest the formation of the triazene intermediate. Therefore, ^Me^CAAC=N_3_−SiMe_3_ remains elusive at room temperature (RT). The formation of dinitrogen was observed by ^14^N NMR spectroscopy.

**Figure 2:**
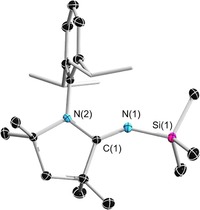
Crystallographically derived molecular structure of ^Me^CAAC=NSiMe_3_. Thermal ellipsoids on the isopropyl groups and all hydrogen atoms have been removed for clarity. Selected bond lengths [Å] and angles [°]: N1−C1 1.2697(8), Si1−N1 1.7000(6), N2−C1 1.3749(8), N2−C4 1.4876(8), C1‐N1‐Si1 145.83(5).

Single‐crystal X‐ray crystallography shows that ^Me^CAAC=NSiMe_3_ (Figure 2) crystallized in the *P*2_1_/*c* space group with four molecules per unit cell. The C(1)−N(1) bond length (1.2697(8) Å) is not significantly different than those of the previously reported *N*‐trimethylsilyl‐2‐iminoimidazolines IMes=NSiMe_3_ (1.267(2) Å) and IDipp=NSiMe_3_ (1.264(2)/1.265(2) Å) (IMes=1,3‐bis(2,4,6‐trimethylphenyl)imidazolin‐2‐ylidene; IDipp=1,3‐bis(2,6‐diisopropylphenyl)imidazolin‐2‐ylidene) whereas the Si(1)−N(1) bond length (1.7000(6) Å) is slightly longer than those of IMes=NSiMe_3_ (1.687(1) Å) and IDipp=NSiMe_3_ (1.677(2) Å).^24^ The C(1)‐N(1)‐Si(1) bond angle 145.83(5)° also falls within the range of the iminoimidazolines. The imine carbon of ^Me^CAAC=NSiMe_3_ has a ^13^C NMR chemical shift of 167.1 ppm, which is much further downfield than those of the *N*‐trimethylsilyl‐2‐iminoimidazolines (139.7 to 144.6 ppm).^24^, ^25^

**Scheme 2:**
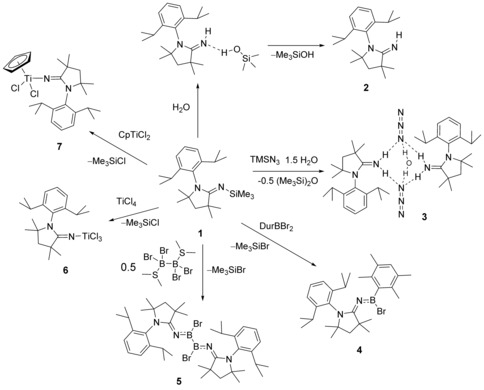
Hydrolysis of ^Me^CAAC=NSiMe_3_ (**1**) and its use as a pyrrolidine‐2‐iminato transfer reagent.

**Figure 3:**
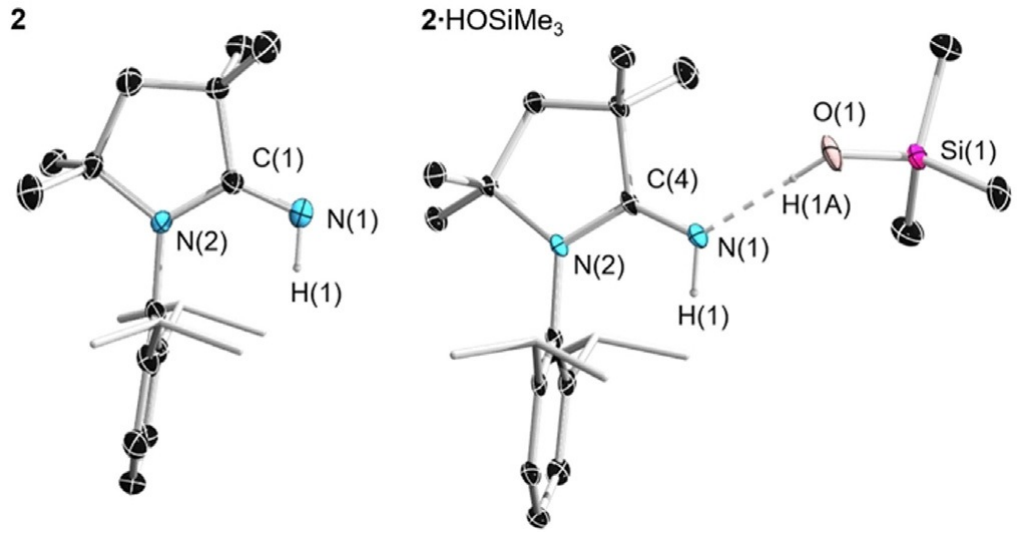
Crystallographically derived molecular structure of ^Me^CAAC=NH (**2**) and ^Me^CAAC=NH⋅HOSiMe_3_ (**2**⋅HOSiMe_3_). Thermal ellipsoids on the isopropyl groups and all hydrogen atoms (except the H1 and H2 atoms) have been removed for clarity. Selected bond lengths [Å] and angles [°] for ^Me^CAAC=NH: N1−C1 1.279(2), N2−C1 1.3734(19), N1‐C1‐N2 128.38(14). For ^Me^CAAC=NH⋅HOSiMe_3_: C4−N1 1.290(3), N1−O1 2.732(4), Si1−O1 1.635(2), Si1−C1 1.879(3), Si1−C2 1.865(3), Si1−C3 1.867(3), N1‐C4‐N2 127.4(2).

It was found that compound **1** was sensitive to moisture, hydrolyzing to ^Me^CAAC=NH (**2**) and trimethylsilanol, which co‐crystallized as the hydrogen‐bonded adduct ^Me^CAAC=NH⋅HOSiMe_3_ (**2**⋅HOSiMe_3_) by slow evaporation of its acetone solution (Scheme 2). The silanol in **2**⋅HOSiMe_3_ appears to be too weakly bound for the acquisition of solution data (i.e. NMR) of the adduct. The trimethylsilanol was easily removed from this adduct by applying a mild vacuum or by sublimation at ca. 60 °C, affording pure ^Me^CAAC=NH. Both MeCAAC=NH⋅HOSiMe_3_ and ^Me^CAAC=NH (Figure 3) were authenticated by X‐ray crystallography. Although hydrogen bonding between Lewis bases and many silanols has been previously observed by X‐ray crystallography,^26^ no such observation has been made with trimethylsilanol. Only the low‐temperature crystal structure of trimethylsilanol has been reported,^27^ making it difficult to compare the Lewis basicity of ^Me^CAAC=NH based on the N−(H)O distance (2.732(4) Å) in ^Me^CAAC=NH⋅HOSiMe_3_. The Si−O distance (1.635(2) Å) is identical to that found in the crystal structure of Me_3_SiOH (1.636(3) Å).^27^

In the crystal structure of ^Me^CAAC=NH (**2**), the imine H atoms are completely isolated from the other molecules and there are no significant intermolecular hydrogen bonds. This is similar to the sterically bulky NHC=NH compounds, whose structures are well isolated, but contrasts with a number of NHC=NH structures that form H‐bonded dimers.^24^ The C(1)−N(1) bond length of **2** (1.279(2) Å) is slightly shorter than that of ^Me^CAAC=NH⋅HOSiMe_3_ (1.290(3) Å) and significantly shorter than those of the NHC=NH compounds (1.294(3)–1.298(1) Å).^24^ This is likely due to the presence of only one competing π‐donating nitrogen group in **2** in contrast with the two π‐donor N atoms of NHC=NH species. It should be mentioned that conventional synthetic approaches to pyrrolidine‐2‐imines require the use of high‐temperature, high‐pressure reactions in stainless‐steel autoclave reactors.^28^ Compounds with the pyrrolidine‐2‐imine skeleton have been shown to be potent inhibitors of nitric oxide synthase,^28^ however, their multistep syntheses involve converting the corresponding lactams to iminoethers with trimethyloxonium tetrafluoroborate in dichloromethane, followed by refluxing with ammonium chloride.^29^ A more convenient, high‐yielding synthesis of pyrrolidine‐2‐imines involved the reaction of γ‐azidonitriles with dichloroindium hydride.^30^ Although CAACs are prepared by multi‐step syntheses and the choice of their substituents is limited, the current reaction of CAACs with azides does provide an alternative synthesis of pyrrolidine‐2‐imines and may be useful in organic synthesis.

**Figure 4:**
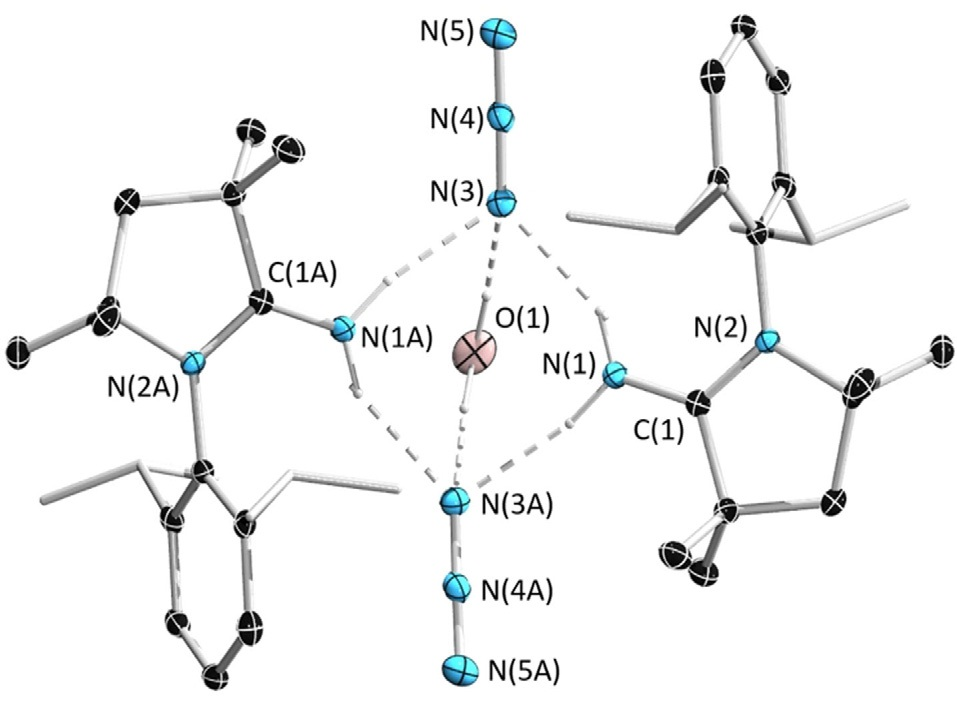
Crystallographically derived molecular structure of ([^Me^CAAC=NH_2_]N_3_)_2_⋅H_2_O (**3**). Thermal ellipsoids on the isopropyl groups and all hydrogen atoms have been removed for clarity except the OH and NH atoms. Selected bond lengths [Å] and angles [°]: N1−C1 1.3037(19), N2−C1 1.3267(18), N3−N4 1.2009(17), N4−N5 1.1663(17), N1(H1)−N3 2.856(2), N1(H2)−N3A 2.879(2), O1(H3)−N3A 2.982(2), N3‐N1‐N(3A) 89.92(5), N(3)‐O(1)‐N(3) 85.60(5).

The hydrolysis of a mixture of ^Me^CAAC=NSiMe_3_ with TMSN_3_ afforded the pyrrolidine iminium azide salt ([^Me^CAAC=NH_2_]N_3_)_2_⋅H_2_O (**3**), which crystallized as a dimer with a molecule of hydrogen‐bonded H_2_O (Figure 4). Shortly after adding water to a mixture of ^Me^CAAC=NSiMe_3_ with TMSN_3_ in C_6_D_6_, a broad signal at 6.3 ppm was observed in the ^1^H NMR spectrum, which is similar to that of neat HN_3_ (6.5 to 6.2 ppm).^31^ An intense singlet at 0.11 ppm in the ^1^H NMR spectrum indicated the formation of O(SiMe_3_)_2_ and suggested complete hydrolysis of TMSN_3_ and ^Me^CAAC=NSiMe_3_. After 12 hours, colorless block‐shaped crystals had formed. The structure of the salt comprises azide anions that are hydrogen‐bonded to both the iminium and water protons. Although various 2‐iminopyrrolidine salts have previously been synthesized, such as 2‐iminopyrrolidine hydrochloride,^29^ no examples of crystal structures of salts containing the 2‐iminopyrrolidine iminium cation could be found in the Cambridge Crystallographic Database.

The crystals of **3** are highly soluble in CDCl_3_ and their ^1^H NMR spectrum contains a broad signal at 6.2 ppm, which integrates to six relative to the dimeric unit, suggesting that the iminium and water protons are in rapid exchange with the azide anions in solution at room temperature. The imine ^13^C NMR signal is at 175.7 ppm, which is downfield relative to **1** (167.1 ppm).

**Figure 5:**
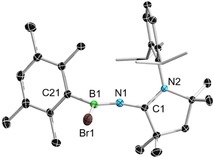
Crystallographically derived molecular structure of ^Me^CAAC=NBBr(Dur) (**4**). Thermal ellipsoids on the isopropyl groups and all hydrogen atoms have been removed for clarity. Selected bond lengths [Å] and angles [°]: N1−C1 1.2705(16), C21−B1 1.574(2), N1−B1 1.338(2), Br1−B1 2.0185(15). N1‐B1‐C2 127.87(12), C1‐N1‐B1 164.20(12), N1‐B1‐Br1 118.02(10), C2‐B1‐Br1 114.01(9).

To investigate the ability of ^Me^CAAC=NSiMe_3_ (**1**) to act as a transfer reagent, its reactions with boron halides were carried out, leading to various aminoborane species by halotrimethylsilane elimination. The overnight reaction of ^Me^CAAC=NSiMe_3_ with one equivalent of DurBBr_2_ (Dur=duryl=2,3,5,6‐tetramethylphenyl) in hexane afforded colorless crystals of ^Me^CAAC=NBBrDur (**4**). The ^11^B NMR spectrum of the crystals showed a significant upfield shift to 27.1 ppm relative to DurBBr_2_ (*δ*=62.7 ppm). The compound was characterized by single‐crystal X‐ray crystallography and the resulting molecular structure is shown in Figure 5. The C(1)−N(1) bond length (1.2705(16) Å) is essentially identical to that of ^Me^CAAC=NSiMe_3_ (1.2697(8) Å), but significantly shorter than that of the structurally similar compound IDipp=NBClPh (1.307(3) Å) in line with the aforementioned shorter exocyclic C=N bonds of CAAC=NR species relative to those of NHC=NR species.^18^ The N(1)−B(1) bond length (1.338(2) Å) is only very slightly shorter than that of IDipp=NBClPh (1.350(3) Å).

**Scheme 3:**
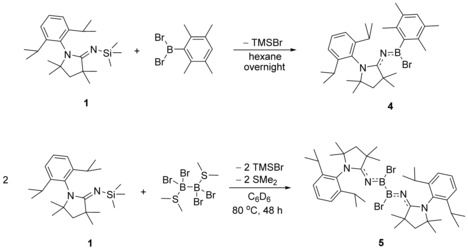
Reactions of ^Me^CAAC=NSiMe_3_ with DurBBr_2_ and B_2_Br_4_⋅SMe_2_.

In order to synthesize a diiminodiborane, ^Me^CAAC=NSiMe_3_ was mixed with half an equivalent of B_2_Br_4_⋅(SMe_2_)_2_ in C_6_D_6_. The reaction solution was stirred at room temperature and monitored by ^1^H and ^11^B NMR spectroscopy, which indicated that no reaction occurred at room temperature even after three days. However, after heating the reaction solution at 80 °C for 48 h, ^1^H NMR spectroscopy showed the formation of TMSBr (0.30 ppm), whereas ^11^B NMR spectroscopy provided a new signal at *δ*=27.9 ppm and the signal attributable to the starting material, B_2_Br_4_⋅(SMe_2_)_2_ (*δ*=−0.3 ppm), had disappeared. This suggested that the reaction had proceeded to completion (Scheme 3).

**Figure 6:**
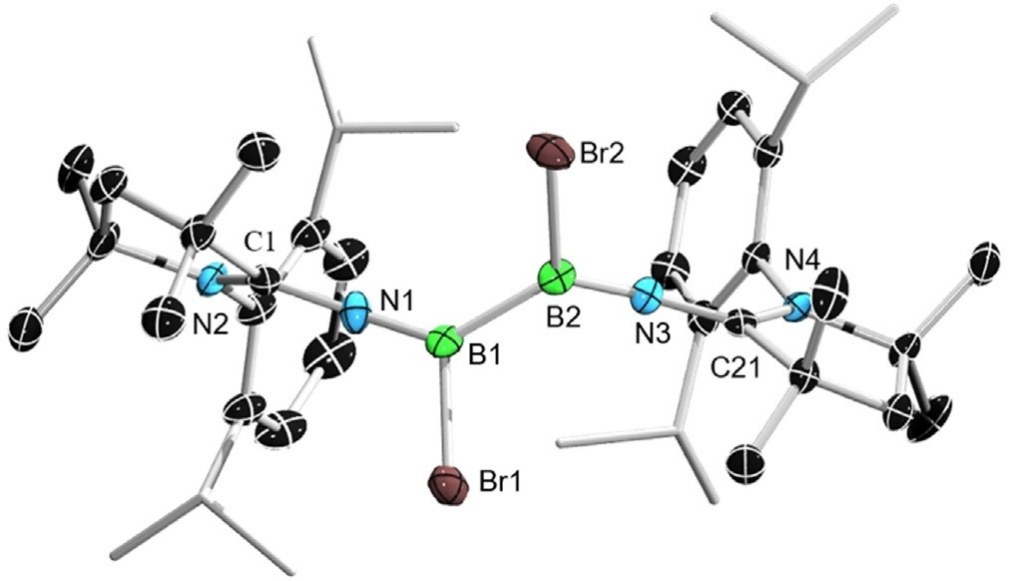
Crystallographically derived molecular structure of compound **5**. Thermal ellipsoids on the isopropyl groups and all hydrogen atoms have been removed for clarity. Selected bond lengths [Å] and angles [°]: B1−B2 1.692(4), N1−B1 1.337(3), N3−B2 1.336(3), C1−N1 1.272(3), C21−N3 1.268(3), Br1−B1 2.036(3), Br2−B2 2.023(3). C1‐N1‐B1 159.3(2), C21‐N3‐B2 162.5(2), N1‐B1‐B2 128.7(2), B1‐B2‐N2 129.5(2), N1‐B1‐Br1 116.83(18), B2‐B1‐Br1 114.45(16), B1‐B2‐Br2 114.17(16), N2‐B2‐Br2 116.34(18).

Colorless crystals of (^Me^CAAC=N)_2_B_2_Br_2_ (**5**) were obtained by slow evaporation of a benzene solution of the compound and were characterized by single‐crystal X‐ray crystallography (Figure 6). The B−N bond length (1.337(3) Å) is equivalent to that of ^Me^CAAC=NBBr(Dur) (1.338(2) Å). The bond lengths fall within the region of B=N double bonds.^32^ The boron centers in both compounds remain in planar environments and the C‐N‐B angles in compound **4** (164.20(12)°) and **5** (C1‐N1‐B1 159.3(2)°, C2‐N2‐B2 162.5(2)°) are only slightly bent. Although compound **5** represents the first crystallographically characterized imino‐substituted diborane, the B−B bond length (1.692(4) Å) is similar to other known dibromodiboranes such as bis(dimethylamino)dibromodiborane, (Me_2_N)B_2_Br_2_ (1.682(16) Å)^33^ and the bis(pyrrolyl)dibromodiborane, (C_4_H_4_N)_2_B_2_Br_2_ (1.696(4) Å).^34^

**Figure 7:**
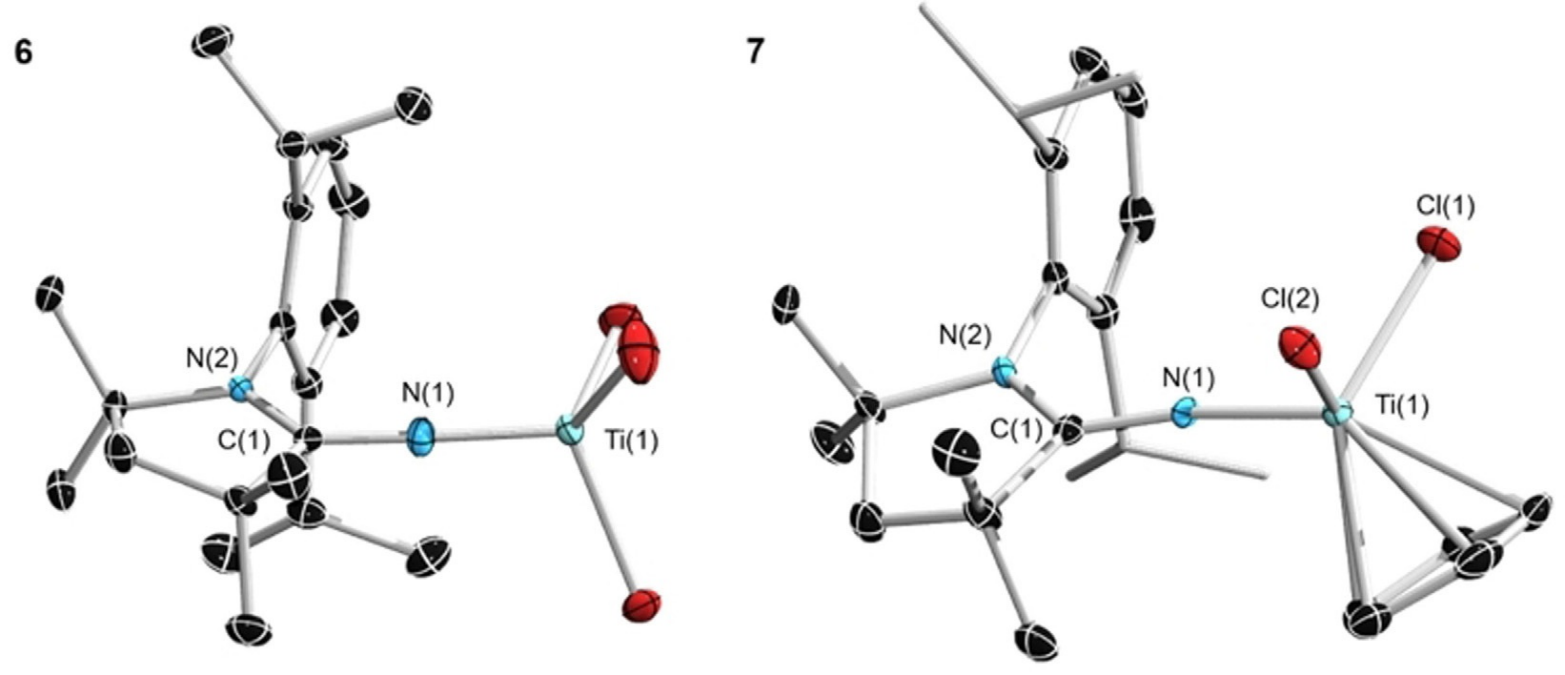
Crystallographically derived molecular structures of **6** and **7**. Hydrogen atoms have been removed for clarity. Selected bond lengths [Å] and angles [°] for **6**: C(1)−N(1) 1.3129(16); C(1)−N(2) 1.3217(17), Ti(1)−N(1) 1.7438(12); Ti−Cl(1) 2.2316(6) Ti(1)−Cl2 2.2411(7); Ti(1)−Cl(3) 2.2285(5); C(1)‐N(1)‐Ti(1) 178.21(11), N(1)‐Ti(1)‐Cl(1) 110.48(4), N(1)‐Ti(1)‐Cl(2) 109.64(5), N(1)‐Ti(1)‐Cl(3) 108.50(4), Cl(3)‐Ti(1)‐Cl(1) 108.27(2), Cl(3)‐Ti(1)‐Cl(2) 109.12(2), Cl(1)‐Ti(1)‐Cl(2) 110.78(2). For **7**: N(1)−C(1) 1.301(7); Ti(1)−N(1) 1.805(4); Ti(1)−Cl(1) 2.3073(18); Ti(1)−Cl(2) 2.2896(18); N(2)−C(1) 1.340(7); C(1)‐N(1)‐Ti(1) 164.3(4); N(1)‐Ti(1)‐Cl(1) 106.10(15); N(1)‐Ti(1)‐Cl(2) 102.78(17); Cl(2)‐Ti(1)‐Cl(1) 101.00(7).

Similar to other N‐heterocyclic carbene adducts of main‐group elements, imidazolin‐2‐iminato ligands have previously been used in transition‐metal catalysis.^35^ Due to their electronic similarity to cyclopentadienyl ligands, Group 4 complexes have been the dominant focus likely due to their use as olefin polymerization catalysts.^25^, ^36^, ^37^, ^38^ Complexes containing imidazolin‐2‐iminato ligands were also shown to catalyze reactions such as the hydroamination and hydrosilylation of alkenes and alkynes,^39^ and alkyne cross‐metathesis.^40^ In order to compare structural parameters and confirm the ability of the ^Me^CAAC=N^−^ anion to act as a ligand for transition metals, titanium complexes were initially targeted. ^Me^CAAC=NSiMe_3_ rapidly reacted with TiCl_4_ in pentane to form a dark orange solution. Evaporation of the volatiles resulted in orange crystals of ^Me^CAAC=NTiCl_3_ (**6**), which were analyzed by single‐crystal X‐ray crystallography. Similarly, the reaction of CAAC=NSiMe_3_ with CpTiCl_3_ in benzene afforded an orange solution, which resulted in orange crystals of ^Me^CAAC=NTiCl_2_Cp (**7**) upon evaporation of the solvent, which were also crystallographically characterized (Figure 7).

The N−Ti bond lengths of ^Me^CAAC=NTiCl_3_ (1.7438(12) Å) and ^Me^CAAC=NTiCl_2_Cp (1.805(4) Å) are both significantly longer than those of the previously characterized imidazoline‐2‐iminato Ti complexes, such as IDipp=NTiCl_3_ (1.731(3) Å) and I*t*Bu=NTiCpCl_2_ (1.765(3) Å; I*t*Bu: 1,3‐di‐*tert*‐butyl‐imidazol‐2‐ylidiene).^25^ Similar to the boron compounds, the C(1)−N(1) distance of **7** 1.301(7) Å is significantly shorter than some of the analogous NHC=NTiCpCl_2_‐containing compounds (1.324(2) and 1.332(4) Å), but in contrast, the bond length is not significantly different than the corresponding bond of IDipp=NTiCpCl_2_ (1.309(2) Å).^25^ In ^Me^CAAC=NTiCl_3_, the C(1)‐N(1)‐Ti(1) angle (178.21(11)°) is close to linearity, whereas in ^Me^CAAC=NTiCl_2_Cp the Ti is bent away from the Dipp substituent of the ^Me^CAAC with a C(1)‐N(1)‐Ti(1) angle of 164.3(4)°. Based on the structural data, it is difficult to predict how these complexes will perform as olefin polymerization catalysts. These studies are currently underway.

## Conclusions

A cyclic (alkyl)(amino)carbene was shown to undergo a Staudinger‐type reaction with TMSN_3_ to yield ^Me^CAAC=NSiMe_3_, which was shown to be an excellent reagent for the transfer of ^Me^CAAC=N^−^ onto both a main‐group element and a transition metal. Hydrolysis yielded the 2‐iminopyrrolidine as the trimethylsiloxane adduct which could be isolated as an intermediate, whereas hydrolysis in the presence of TMSN_3_ yielded the pyrrolidinium azide salt. ^Me^CAAC=N^−^ ligands were successfully installed as anionic substituents onto a borane and a diborane. The ^Me^CAAC=NSiMe_3_ reagent was also used to prepare pyrrolidine‐2‐iminato Ti complexes, which are potential olefin polymerization precatalysts. Similar to the family of imidazoline‐2‐iminato ligands, we anticipate that this class of ligands will find widespread use in both transition metal and main‐group chemistry. We are currently exploring the reaction of ^Me^CAAC with different covalent azides and the use of the pyrrolidine‐2‐iminato ligand in the stabilization of reactive boron species.^42^

## Experimental Section

**General considerations**: All reactions were performed under an atmosphere of dry argon using standard Schlenk or glovebox techniques unless noted. ^1^H, ^11^B, ^13^C, and ^29^Si NMR spectra were obtained at ambient temperature using a Bruker Avance I 400 (operating at 400 MHz for ^1^H, 128 MHz for ^11^B, 101 MHz for ^13^C, 79.5 MHz for ^29^Si, and 28.9 MHz for ^14^N). ^1^H NMR spectra were referenced to the residual proton resonances of C_6_D_6_ (^1^H, 7.16 ppm) or CDCl_3_ (^1^H, 7.26 ppm). ^13^C NMR spectra were referenced to C_6_D_6_ (^13^C, 128.06 ppm) or CDCl_3_ (^13^C, 77.16 ppm). High‐resolution mass spectrometry data were obtained from a Thermo Scientific Exactive Plus spectrometer in LIFDI (liquid injection field desorption ionization) mode for air‐sensitive samples or ASAP (atmospheric solids analysis probe) for air‐stable compounds. All solvents were purified by distillation using the appropriate drying agents (potassium/benzophenone for diethyl ether; Na/K alloy for pentane and hexane; sodium for toluene, benzene), deoxygenated using three freeze‐pump‐thaw cycles and stored over molecular sieves under dry argon prior to use. Deuterated solvents used for NMR spectroscopy were purchased from Sigma–Aldrich, deoxygenated by three freeze‐pump‐thaw cycles and dried under an argon atmosphere over molecular sieves. Trimethylsilyl azide was purchased from Sigma–Aldrich. ^Me^CAAC^41^ was prepared by established methods.

**Preparation of** ^**Me**^ **CAAC=NSiMe_3_ (1)**: To a solution of ^Me^CAAC (300 mg, 1.04 mmol, 1 equiv) in pentane (ca. 10 mL) was added a solution of trimethylsilyl azide (160 mg, 2.09 mmol, 2 equiv) in pentane (ca. 5 mL). The reaction was left to stir for 48 h at room temperature, gas release from the glass vessel was observed during this period. After removing all the solvent in vacuo, ^Me^CAAC=NSiMe_3_ (**1**) was obtained as a white solid (318 mg, 0.853 mmol, 82 %). In alternative, more rapid syntheses, hexane was used as a solvent and the reaction mixture was heated to 50–60 °C for 1 hour. ^1^H NMR (400 MHz, C_6_D_6_, 297 K) *δ*=7.19 (t, 1 H, ^3^ *J*=7.3 Hz, CH(Ar‐Dipp)), 7.12 (d, 2 H, ^3^ *J*=7.3 Hz, CH(Ar‐Dipp)), 3.08 (sept, 2 H, ^3^ *J*=6.8 Hz, CH(*i*Pr‐Dipp)), 1.76 (s, 2 H, CH_2_), 1.27 (d, 6 H, ^3^ *J*=6.8 Hz, CH_3_(*i*Pr‐Dipp)), 1.24 (d, 6 H, ^3^ *J*=6.8 Hz, CH_3_(*i*Pr‐Dipp)), 1.21 (s, 6 H, CAAC‐C(CH_3_)_2_), 1.01 (s, 6 H, CAAC‐C(CH_3_)_2_), 0.23 ppm (s, 9 H, (CH_3_)_3_Si). ^13^C{^1^H} NMR (101 MHz, C_6_D_6_, 297 K) *δ*=167.1 (C=N), 149.4 (Ar‐Dipp), 133.8 (*p*‐Ar‐Dipp), 123.8 (*m*‐Ar‐Dipp), 59.5 (CAAC‐NC(CH_3_)_2_), 52.5 (CAAC‐CH_2_), 41.5 (CAAC‐C(CH_3_)_2_), 29.8 (CAAC‐C(CH_3_)_2_), 29.1 (CH(*i*Pr‐Dipp)), 29.0 (CAAC‐C(CH_3_)_2_), 26.7 (CH_3_(*i*Pr‐Dipp)), 23.1 (CH_3_(*i*Pr‐Dipp)), 3.9 ppm ((CH_3_)_3_Si). ^29^Si NMR (79.5 MHz, C_6_D_6_, 297 K) *δ*=−17.7 ppm. LIFDI‐MS: *m*/*z*: calcd for C_23_H_40_N_2_Si: 372.2961; found: 372.2955.

**Preparation of** ^**Me**^ **CAAC=NH (2) and** ^**Me**^ **CAAC=NH⋅HOSiMe_3_ (2⋅HOSiMe_3_)**: Undried acetone was added to ^Me^CAAC=NSiMe_3_ (20 mg, 0.054 mmol). The resulting solution was left to slowly evaporate under air. Colorless crystals of ^Me^CAAC=NH⋅HOSiMe_3_ (19 mg, 0.048 mmol, 90 % yield) were obtained after leaving the oily residue to stand for several days. The trimethylsiloxane slowly dissociated from the crystals upon standing in the open air for three days, which was determined by ^1^H NMR spectroscopy. To obtain pure ^Me^CAAC=NH, the crystals were sublimed at 80 °C under ambient pressure to yield colorless rod‐shaped crystals (14 mg, 0.047 mmol, 88 % yield). ^1^H NMR (400 MHz, C_6_D_6_, 297 K) *δ*=7.31 (t, 1 H, ^3^ *J*=7.6 Hz, CH(Ar‐Dipp)), 7.22 (d, 2 H, ^3^ *J*=7.6 Hz, CH(Ar‐Dipp)), 5.0 (s, broad, 1 H, N‐H), 3.08 (sept, 2 H, ^3^ *J*=6.8 Hz, CH(*i*Pr‐Dipp)), 1.87 (s, 2 H, CH_2_), 1.51 (s, 6 H, CAAC‐C(CH_3_)_2_), (m, 12 H, ^3^ *J*=7.78 Hz, CH_3_(*i*Pr‐Dipp)), 1.12 ppm (s, 6 H, CAAC‐C(CH_3_)_2_). ^13^C{^1^H} NMR (101 MHz, C_6_D_6_, 297 K) *δ*=173.9 (C=N), 150.6 (Ar‐Dipp), 129.1 (Ar‐Dipp), 124.8 (Ar‐Dipp), 61.6 (CAAC‐NC(CH_3_)_2_), 52.4 (CAAC‐CH_2_), 40.8 (CAAC‐C(CH_3_)_2_), 29.9 (CH(*i*Pr‐Dipp)), 29.5 (CH(*i*Pr‐Dipp)), 29.0 (CH(*i*Pr‐Dipp)), 26.6 (CH_3_(*i*Pr‐Dipp), 23.3 ppm (C(CH_3_)_2_). ^1^H NMR (400 MHz, CDCl_3_, 297 K) *δ*=7.35 (t, 1 H, ^3^ *J*=7.6 Hz, CH(Ar‐Dipp)), 7.24 (d, 2 H, ^3^ *J*=7.6 Hz, CH(Ar‐Dipp)), 3.01 (sept, 2 H, ^3^ *J*=6.8 Hz, CH(*i*Pr‐Dipp), 2.07 (s, 2 H, CH_2_), 1.42 (s, 6 H, CAAC‐C(CH_3_)_2_)), 1.24 (d, 6 H, ^3^ *J*=6.8 Hz, CH_3_(*i*Pr‐Dipp)), 1.20 (s, 6 H, (CAAC‐C(CH_3_)_2_)), 1.08 ppm (d, 6 H, ^3^ *J*=6.7 Hz, CH_3_(*i*Pr‐Dipp)). ^13^C{^1^H} NMR (101 MHz, CDCl_3_, 297 K): 174.6 (C=NH), 150.4 (C‐Ar), 130.5 (C‐Ar), 129.0 (C‐Ar), 124.7 (C‐Ar), 62.4 (NC(CH_3_)_2_), 52.4 (CAAC‐CH_2_), 40.9 (CAAC‐C(CH_3_)_2_), 29.7 (Dipp(C(CH_3_)_2_)), 29.6 (Dipp(C(CH_3_)_2_)), 28.8 CAAC‐(C(CH_3_)_2_), 26.7 Dipp(C(CH_3_)_2_), 23.3 CAAC‐(C(CH_3_)_2_), 1.6 ppm (silicone grease). ASAP‐MS: *m*/*z*: calcd for C_20_H_32_N_2_: 301.2643; found: 301.2627 [*M*+H]^+^.

**Preparation of ([^Me^CAAC=NH_2_]N_3_)_2_⋅H_2_O (3)**: A mixture of ^Me^CAAC (24 mg, 0.084 mmol) and excess TMSN_3_ (35 mg, 0.30 mmol) were mixed in C_6_D_6_ inside a NMR tube with a Teflon seal and the mixture was heated to 80 °C for 1 h. Excess water (12 mg, 0.55 mmol) was syringed into the NMR tube, which caused bubbles to form around the water droplet. The NMR tube was heated to 60 °C for 10 min and then allowed to sit at room temperature overnight, during which time, clear, colorless, block‐shaped crystals of ([^Me^CAAC=NH_2_]N_3_)_2_⋅H_2_O formed. Volatiles were removed under dynamic vacuum, which resulted in the formation of additional white solid. Isolated yield: 28 mg, 93 %. ^1^H NMR (400 MHz, CDCl_3_, 297 K) *δ*=7.52 (t, 1 H, ^3^ *J*=7.7 Hz, CH(Ar‐Dipp)), 7.35 (d, 2 H, ^3^ *J*=7.7 Hz, CH(Ar‐Dipp)), 6.20 (s, broad, 3 H, NH_2_/0.5H_2_O), 2.71 (sept, 2 H, ^3^ *J*=6.6 Hz, (CH(*i*Pr‐Dipp))), 1.62 (s, 6 H, CAAC‐(C(CH_3_)_2_)), 1.32 (s, 6 H, CAAC‐(C(CH_3_)_2_)), 1.27 (d, 6H ^3^ *J*=6.6 Hz, (CH_3_(*i*Pr‐Dipp))), 1.16 ppm (d, 6 H, ^3^ *J*=6.6 Hz, (CH_3_(*i*Pr‐Dipp))). ^13^C{^1^H} NMR (101 MHz, CDCl_3_, 297 K) *δ*=175.7 (C=N ring), 147.38 (Ar‐C), 131.68 (Ar‐C), 126.1 (Ar‐C), 125.8 (Ar‐C), 69.7 C‐N, 51.2 (CH_2_), 42.8 (ring‐C(CH_3_)_2_), 29.3 (CH_3_), 29.0 (CH(*i*Pr‐Dipp)), 28.1 (CH(*i*Pr‐Dipp)), 25.9 (CH_3_(*i*Pr‐Dipp), 23.5 ppm (CH_3_(*i*Pr‐Dipp). Signals due to residual C_6_D_6_ at 128.1, 127.9, and 127.6 ppm. ^14^N NMR (28.9 MHz, CDCl_3_, 297 K) *δ*=267, 204, 62 ppm. ASAP‐MS: *m*/*z*: calcd for C_20_H_33_N_2_: 301.2644; found: 301.2626.

**Preparation of** ^**Me**^ **CAAC=NBBr(Dur) (4)**: To a solution of duryldibromoborane (12 mg, 0.04 mmol, 1 equiv) in hexane (2 mL) was added a solution of ^Me^CAAC=NSiMe_3_ (**1**, 15 mg, 0.04 mmol, 1 equiv) in hexane (1 mL). The reaction solution was left to stand overnight at room temperature, at which point colorless crystals formed. After decanting all the solvent, the crystals were dried in vacuo to yield ^Me^CAAC=NBBrDur (**4**) as a white solid, a second crop of **4** was obtained by recrystallization from the supernatant at −25 °C (15 mg, 0.028 mmol, 71 %). ^1^H NMR (400 MHz, C_6_D_6_, 297 K) *δ*=7.18 (t, 1 H, ^3^ *J*=7.8 Hz, CH(Ar‐Dipp) overlapped with C_6_D_6_), 7.08 (d, 2 H, ^3^ *J*=7.8 Hz, CH(Ar‐Dipp)), 6.82 (s, 1 H, CH(Ar‐Dur)), 2.97 (br, 2 H, CH(*i*Pr‐Dipp)), 2.07 (s + br, 12 H, CH_3_‐Dur), 1.68 (s, 2 H, CH_3_‐Dur), 1.39 (s, 6 H, CAAC‐C(CH_3_)_2_), 1.13 (d + br, 12 H, unresolved *J*, CH_3_(*i*Pr‐Dipp)), 0.97 ppm (s, 6 H, CH_3_(*i*Pr‐Dipp)). ^13^C{^1^H} NMR (101 MHz, C_6_D_6_, 297 K) *δ*=161.5 (C=N), 149.0 (br, Ar‐Dipp), 133.8 (br, B‐C‐Dur), 132.8 (*m*‐Ar‐Dur), 132.1 (*o*‐Ar‐Dur), 131.1 (*p*‐Ar‐Dur), 129.1 (*p*‐Ar‐Dipp), 124.9 (*m*‐Ar‐Dipp), 63.6 (CAAC‐NC(CH_3_)_2_), 51.1 (CAAC‐CH_2_), 43.0 (CAAC‐NCC(CH_3_)_2_), 29.0 (CAAC‐C(CH_3_)_2_), 28.6 (br, CAAC‐C(CH_3_)_2_ + CH(*i*Pr‐Dipp)), 26.4 (br), 24.1 (CH_3_(*i*Pr‐Dipp)), 19.8 ppm (CH_3_‐Dur). ^11^B NMR (128 MHz, CDCl_3_, 297 K) *δ*=27.1. LIFDI‐MS: *m*/*z*: calcd for C_30_H_44_BBrN_2_: 522.2781; found: 522.2731.

**Preparation of (^Me^CAAC=N)_2_B_2_Br_2_ (5)**: To a solution of B_2_Br_4_⋅(SMe_2_)_2_ (19 mg, 0.040 mmol, 0.5 equiv) in C_6_D_6_ was added a solution of **1** (30 mg, 0.08 mmol, 1 equiv) in C_6_D_6_. The reaction solution was heated to 80 °C and monitored by ^1^H and ^11^B NMR spectroscopy. Product **5** was obtained from slow evaporation of C_6_D_6_ as colorless crystals (24 mg, 0.031 mmol, 76 %). Although the yield shown is not quantitative, no other undesired byproducts were observed and therefore, the reaction appears quantitative. For a larger scale synthesis, benzene was added to a Schlenk flask charged with **1** (80 mg, 0.214 mmol, 1 equiv) and B_2_Br_4_⋅(SMe_2_)_2_ (50.0 mg, 0.107 mmol, 0.5 equiv). The mixture was heated to 60 °C for 48 h, and volatiles were removed under dynamic vacuum. After washing the resulting solid with cold pentane and drying under vacuum, **5** was obtained as a white solid. Colorless crystals of **5** were obtained by slow evaporation of a benzene solution. Yield: 62 mg, 74 %. ^1^H NMR (400 MHz, C_6_D_6_, 297 K) *δ*=7.26 (t, 2 H, ^3^ *J*=7.7 Hz, CH(Ar‐Dipp)), 7.08 (d, 4 H, ^3^ *J*=7.7 Hz, CH(Ar‐Dipp)), 3.00 (br, 4 H, CH(*i*Pr‐Dipp)), 1.65 (s, 4 H, CH_2_), 1.47 (br, 24 H, CH_3_(*i*Pr‐Dipp)+CAAC‐C(CH_3_)_2_), 1.19 (d, 12 H, ^3^ *J*=6.8 Hz, CH_3_(*i*Pr‐Dipp)), 0.93 ppm (s, 12 H, CAAC‐C(CH_3_)_2_). ^13^C{^1^H} NMR (101 MHz, C_6_D_6_, 297 K) *δ*=160.40 (C=N), 149.3 (Ar‐Dipp), 131.5 (Ar‐Dipp), 128.5 (*p*‐Ar‐Dipp), 124.4 (*m*‐Ar‐Dipp), 63.4 (CAAC‐NC(CH_3_)_2_), 51.1 (CAAC‐CH_2_), 43.3 (CAAC‐NCC(CH_3_)_2_), 29.4 (br, CH_3_(*i*Pr‐Dipp)+CAAC‐C(CH_3_)_2_), 29.1 (s+br, CH(*i*Pr‐Dipp)+CAAC‐C(CH_3_)_2_), 23.5 ppm (CH_3_(*i*Pr‐Dipp)). ^11^B NMR (128 MHz, C_6_D_6_, 297 K) *δ*=27.4. LIFDI‐MS: *m*/*z*: calcd for C_40_H_62_B_2_Br_2_N_4_: 778.3527; found: 683.4429. This signal matches a formula of C_40_H_66_BrN_4_ (683.4445), which would correspond to two CAAC units, one Br and N_2_H_4_, however, there is no obvious rational explanation for this fragmentation pattern.

**Preparation of** ^**Me**^ **CAAC=NTiCl_3_ (6)**: A solution of ^Me^CAAC=NSiMe_3_ (20 mg, 0.054 mmol) in pentane was slowly added to a large excess of TiCl_4_ (82 mg, 0.43 mmol) in pentane at 0 °C. The solution instantly turned dark orange. Evaporation of the volatiles resulted in the formation of dark orange crystalline solid which were then recrystallized from pentane to form yellow crystals. Yield 20 mg, 82 %. ^1^H NMR (400 MHz, C_6_D_6_, 297 K) *δ*=7.19 (t, 1 H, ^3^ *J*=7.6 Hz, CH(Ar‐Dipp)), 7.05 (d, 2 H, ^3^ *J*=7.6 Hz, CH(Ar‐Dipp)), 2.66 (sept, 2 H, ^3^ *J*=6.5 Hz, CH(*i*Pr‐Dipp)), 1.47 (d, 6 H, ^3^ *J*=6.7 Hz, CH_3_(*i*Pr‐Dipp)), 1.39 (s, 2 H, CH_2_), 1.32 (s, 6 H, CAAC‐C(CH_3_)_2_), 1.07 (d, 6 H, ^3^ *J*=6.7 Hz, CH_3_(*i*Pr‐Dipp)), 0.73 ppm (s, 6 H, CAAC‐C(CH_3_)_2_). ^13^C NMR (101 MHz, C_6_D_6_, 297 K) *δ*=175.3 (C=N), 146.9 (Ar‐C), 130.7 (Ar‐C), 128.9 (Ar‐C), 125.5 (*m*‐Ar‐Dipp), 67.2 (CAAC‐NC(CH_3_)_2_), 49.8 (CAAC‐CH_2_), 47.0 (CAAC‐C(CH_3_)_2_), 29.6 (CAAC‐C(CH_3_)_2_), 28.5 (CH(*i*Pr‐Dipp)), 28.3 (CH(*i*Pr‐Dipp)), 27.0 (CH_3_(*i*Pr‐Dipp)), 23.6 ppm (CH_3_(*i*Pr‐Dipp)). LIFDI‐MS: *m*/*z*: calcd for C_20_H_31_Cl_3_N_2_Ti: 452.1032; found: 301.2629 (only [CAAC=NH_2_]^+^ detected).

**Preparation of (^Me^CAAC=N)(Cp)TiCl_2_ (7)**: A solution of ^Me^CAAC=NSiMe_3_ (17 mg, 0.046 mmol) in benzene was added to a yellow solution of CpTiCl_3_ (10 mg, 0.046 mmol) in benzene and resulted in an orange solution. The mixture was stirred overnight and the solvent was allowed to slowly evaporate. Dark orange block‐shaped crystals formed in a quantitative yield and no purification was required. Yield: 21 mg, 93 %. ^1^H NMR (400 MHz, C_6_D_6_, 297 K) *δ*=7.19 (t, 1 H, ^3^ *J*=7.3 Hz, CH(Ar‐Dipp)) overlapped with solvent, 7.10 (d, 2 H, ^3^ *J*=7.6 Hz, CH(Ar‐Dipp)), 6.23 (s, 5 H, Cp), 2.82 (sept, 2 H, ^3^ *J*=6.9 Hz, CH(*i*Pr‐Dipp)), 1.54 (s, 2 H, (CH_2_), 1.48 (d, 6 H, ^3^ *J*=6.7 Hz CH_3_(*i*Pr‐Dipp)), 1.35 (s, 6 H, CAAC‐C(CH_3_)_2_), 1.13 (d, 6 H, ^3^ *J*=6.8 Hz, CH_3_(*i*Pr‐Dipp)), 0.86 ppm (s, 6 H, CAAC‐C(CH_3_)_2_). ^13^C{^1^H} NMR (101 MHz, C_6_D_6_, 297 K) *δ*=173.9 (C=N), 147.8 (Ar‐C), 131.0 (Ar‐C), 129.8 (Ar‐C), 125.17, 116.0 (Cp), 64.94, 51.09, 46.75, 29.30, 29.07, 28.62, 26.60, 24.18 ppm. LIFDI‐MS: *m*/*z*: calcd for C_26_H_39_Cl_2_N_2_Ti: 497.1970; found: 482.1726 [M−CH_3_]^+^.

## Conflict of interest

The authors declare no conflict of interest.

## Supporting information

As a service to our authors and readers, this journal provides supporting information supplied by the authors. Such materials are peer reviewed and may be re‐organized for online delivery, but are not copy‐edited or typeset. Technical support issues arising from supporting information (other than missing files) should be addressed to the authors.

Supplementary
