# Supplementary material for: MeCAAC=N−: A Cyclic (Alkyl)(Amino)Carbene Imino Ligand
Source: Chemistry. 2020 Jan 9;26(5):1136–43. doi: 10.1002/chem.201904715 (PMC7027825; doi:10.1002/chem.201904715)
Supplement: Supplementary file 1 — Supplementary [file CHEM-26-1136-s001.pdf]

CHEMISTRY
A European Journal

Supporting Information

^{Me}CAAC=N^{-}: A Cyclic (Alkyl)(Amino)Carbene Imino Ligand

James T. Goettel,[a, b] Haopeng Gao,[a, b] Simon Dotzauer,[a, b] and Holger Braunschweig*[a, b]

chem_201904715_sm_miscellaneous_information.pdf

NMR Spectra

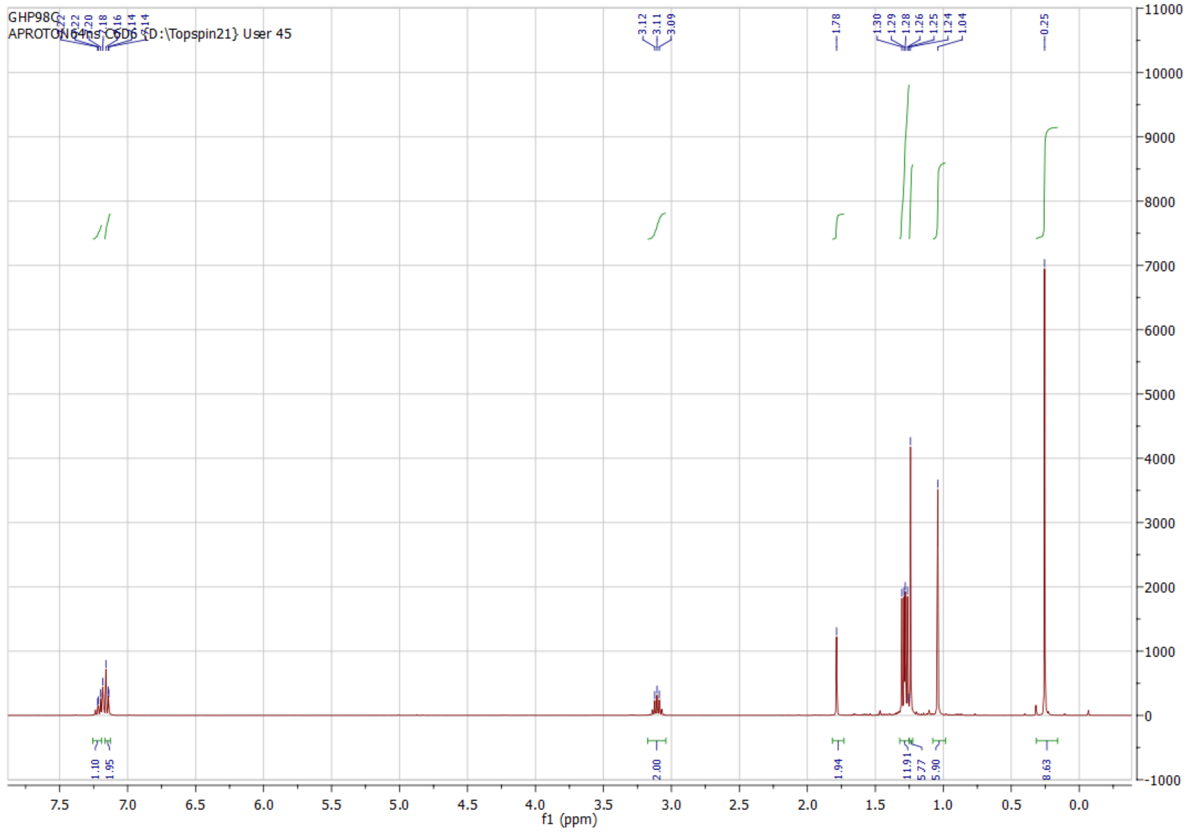

Figure S1. ^{1}H NMR (400 MHz, C_6D_6) spectrum of $^{Me}CAAC{=}NSiMe_3$ (**1**)

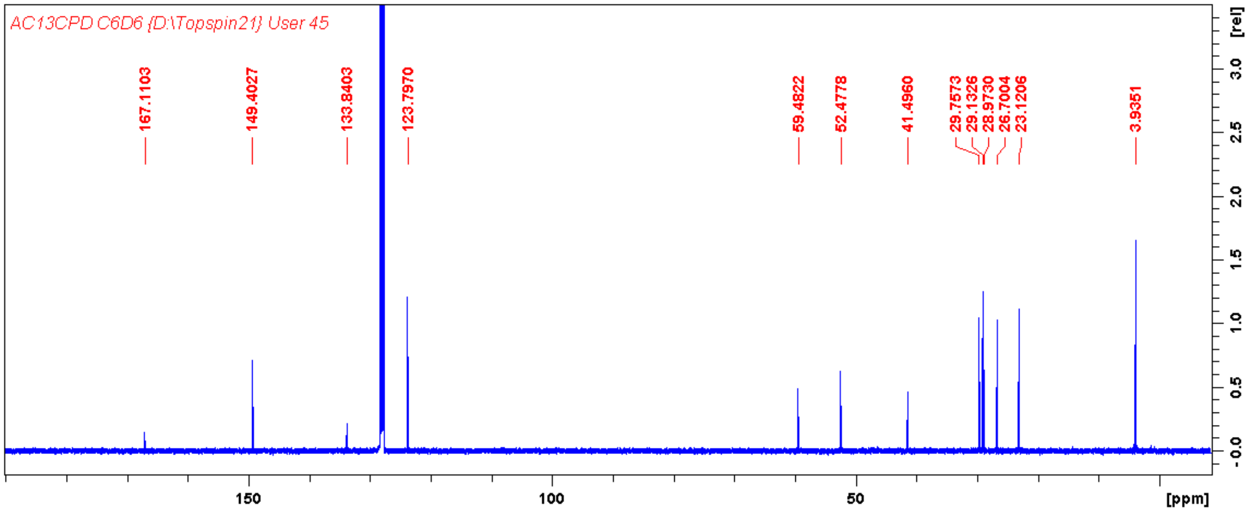

Figure S2. $^{13}C\{^{1}H\}$ NMR (100 MHz, C_6D_6) spectrum of $^{Me}CAAC{=}NSiMe_3$ (**1**)

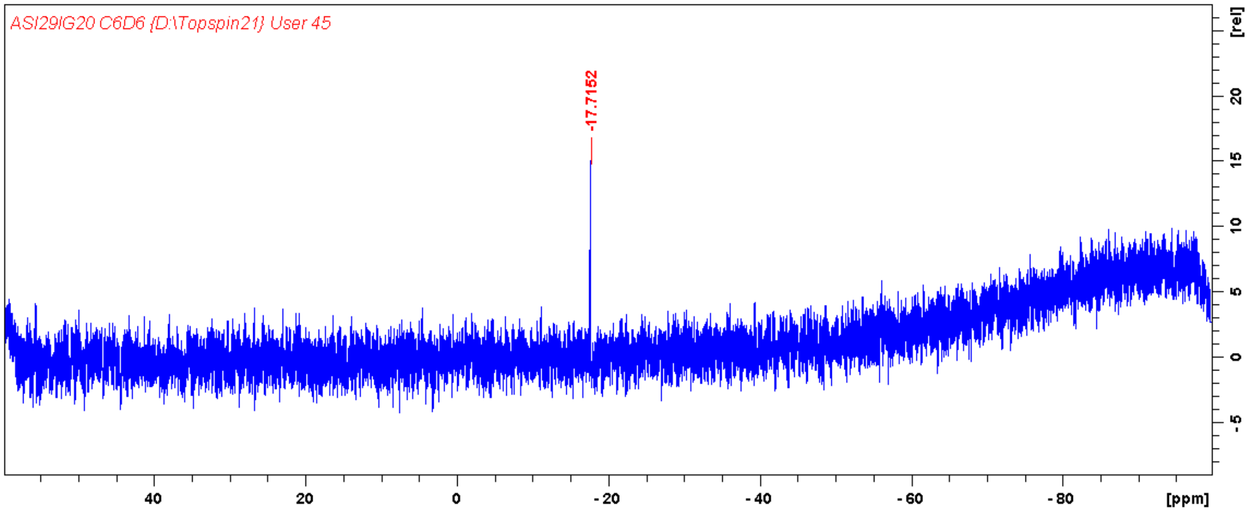

Figure S3. ^{29}Si NMR (79 MHz, C_6D_6) spectrum of ^{Me}CAAC=NSiMe$_3$ (**1**)

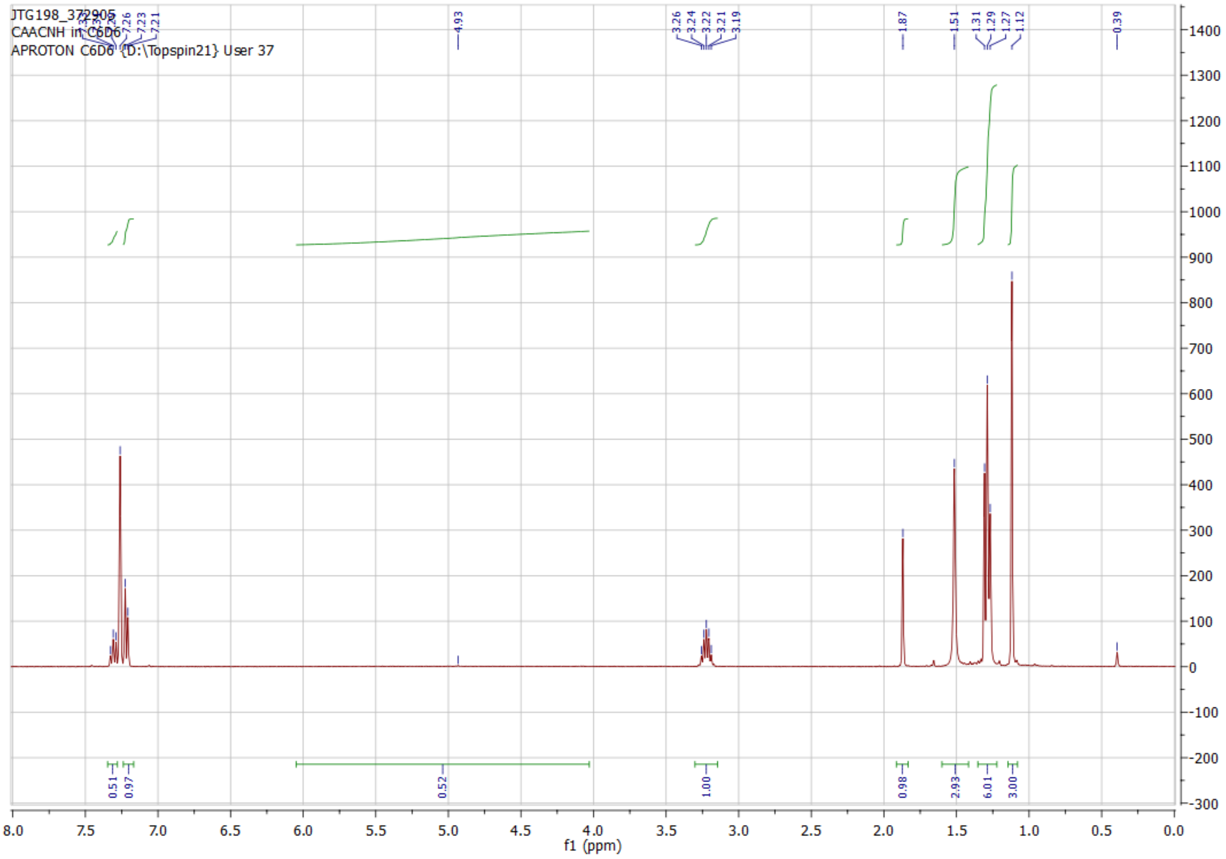

Figure S4. ^{1}H NMR (400 MHz, C_6D_6) spectrum of ^{Me}CAAC=NH (**2**)

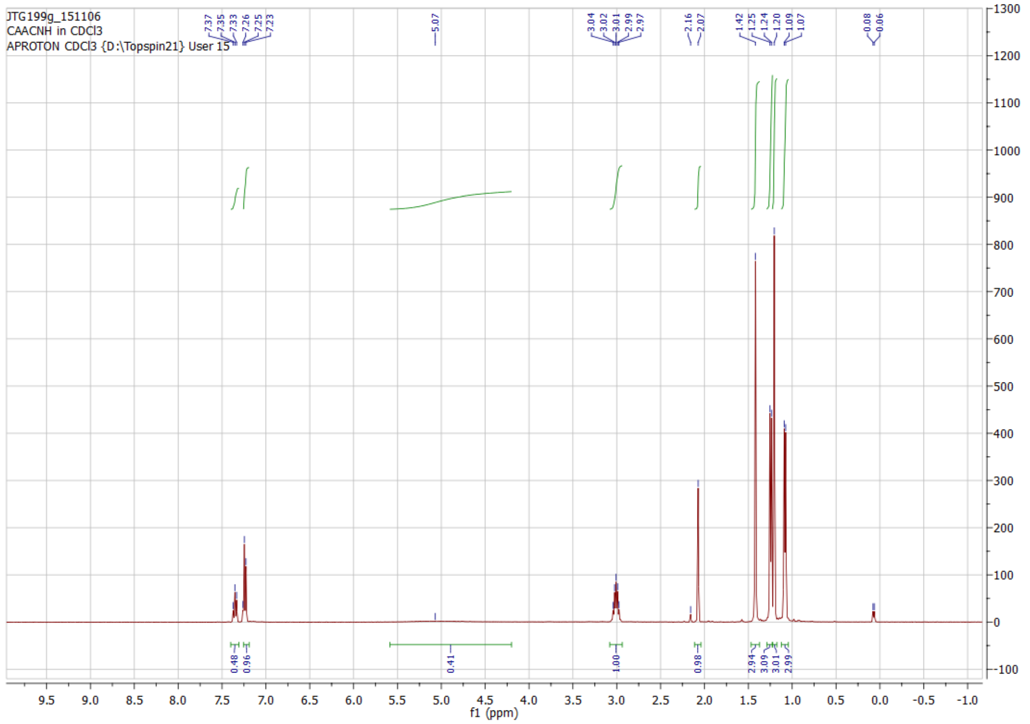

Figure S5. ^{1}H NMR (400 MHz, $CDCl_3$) spectrum of ^{Me}CAAC=NH (**2**)

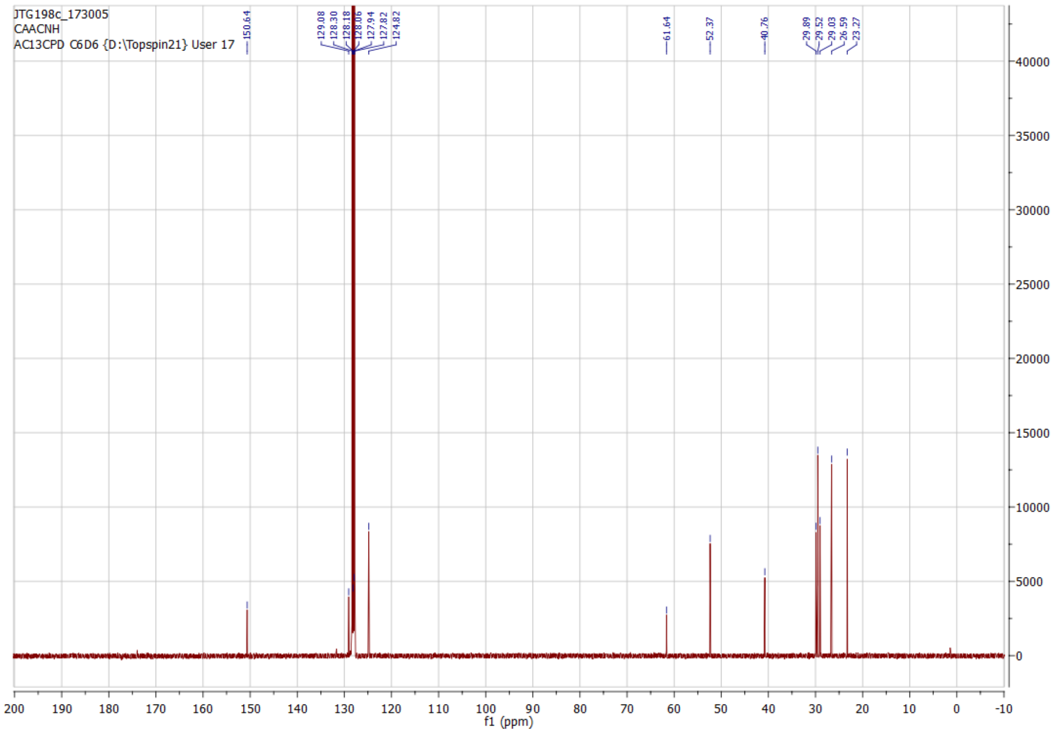

Figure S6. $^{13}C\{^{1}H\}$ NMR (100 MHz, C_6D_6) spectrum of ^{Me}CAAC=NH (**2**)

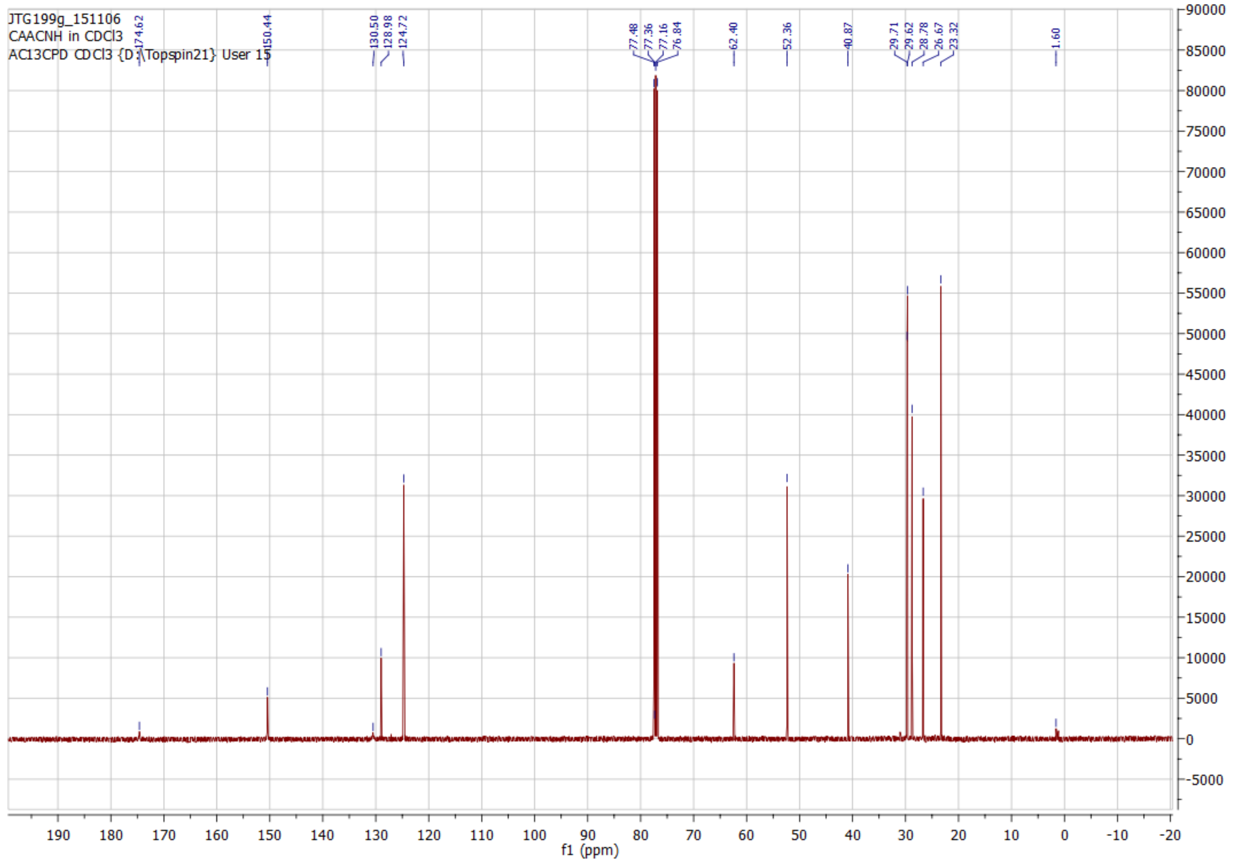

Figure S7. $^{13}C\{^{1}H\}$ NMR (100 MHz, $CDCl_3$) spectrum of ^{Me}CAAC=NH (**2**)

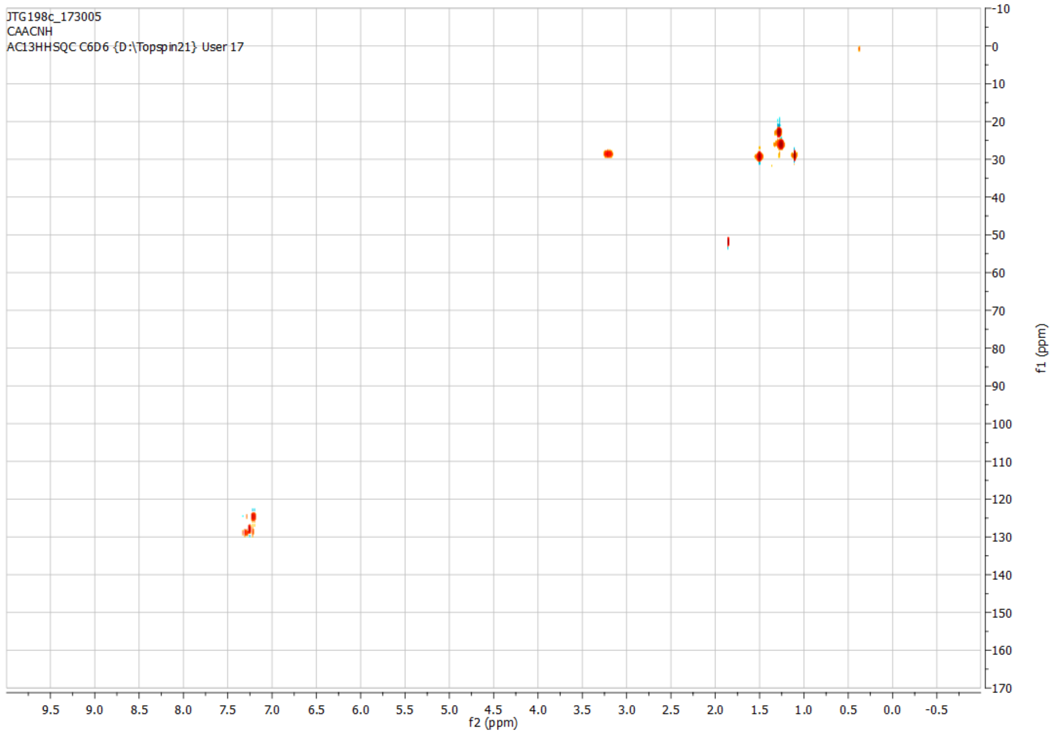

Figure S8. $^{13}C\{^{1}H\}$ NMR (100 MHz, C_6D_6) spectrum of ^{Me}CAAC=NH (**2**)

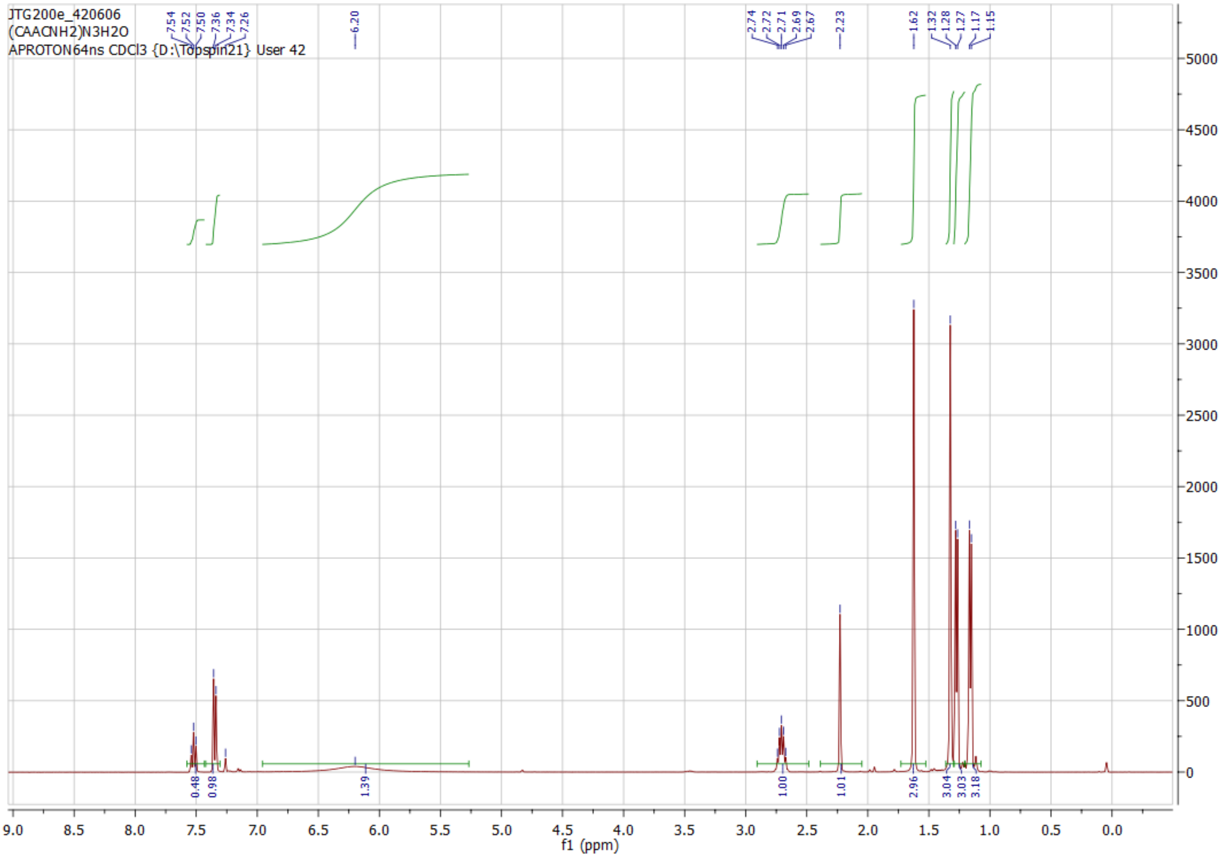

Figure S9. ^{1}H NMR (400 MHz, $CDCl_3$) spectrum of ($[^{Me}CAAC{=}NH_2]N_3)_2 \cdot H_2O$ (**3**)

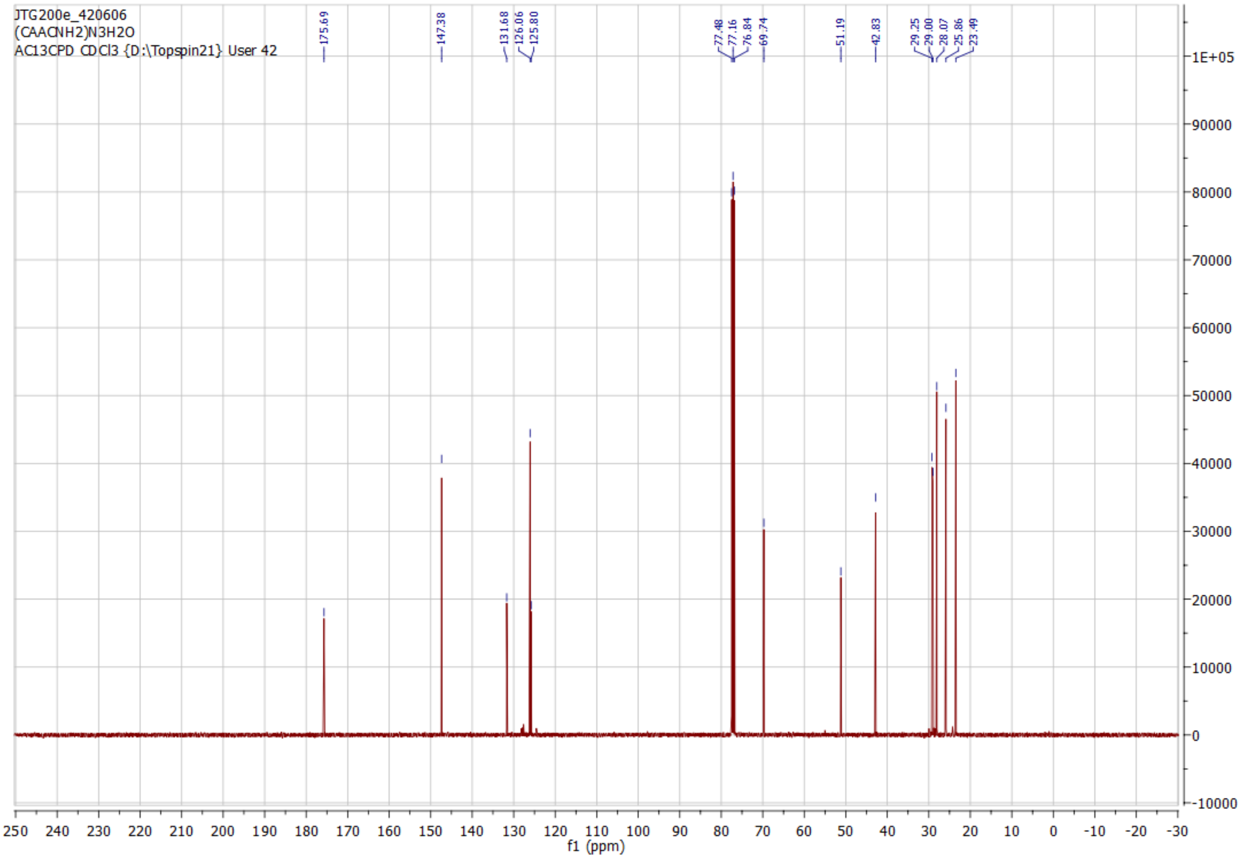

Figure S10. $^{13}C\{^{1}H\}$ NMR (100 MHz, $CDCl_3$) spectrum of ($[^{Me}CAAC{=}NH_2]N_3)_2{\cdot}H_2O$ (**3**)

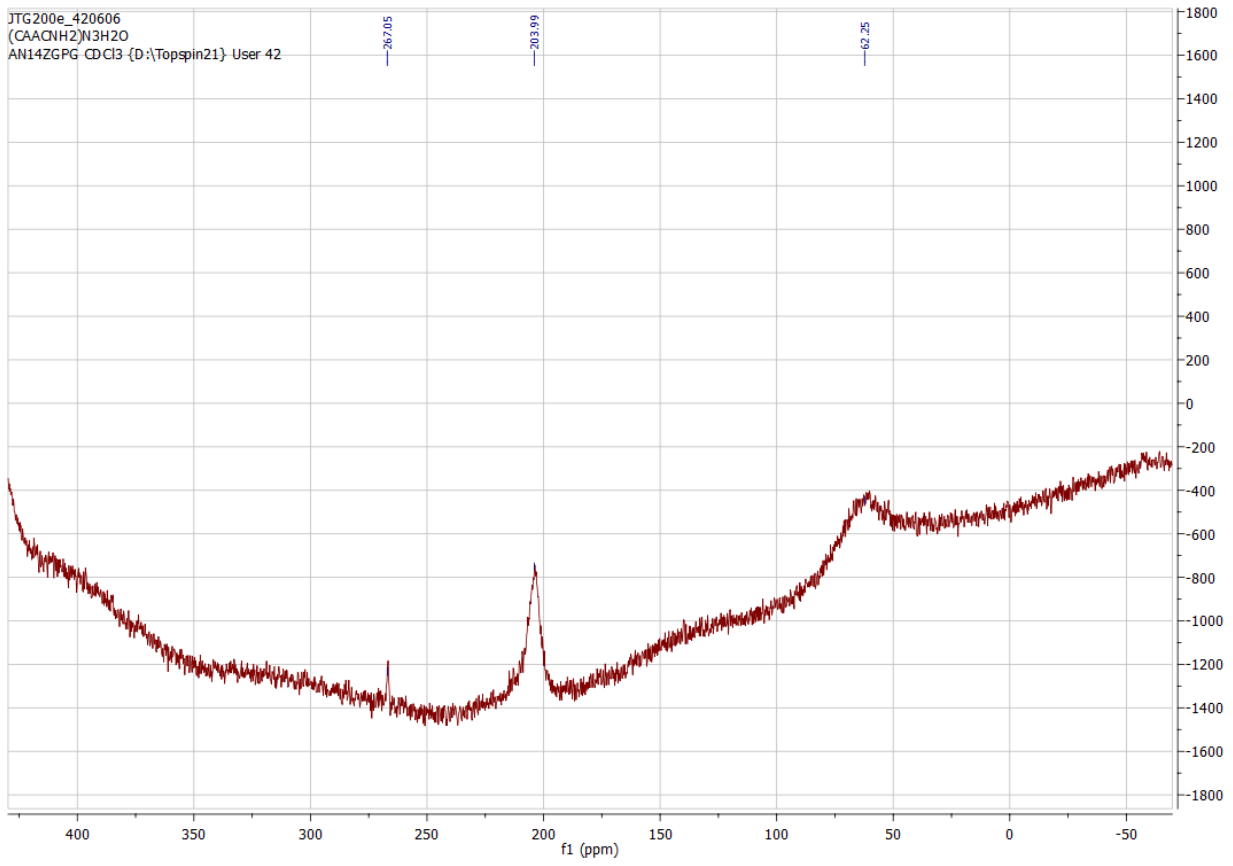

Figure S11. ^{14}N NMR (26 MHz, $CDCl_3$) spectrum of ([^{Me}CAAC=NH_2]N_3)$_2$·H_2O (**3**)

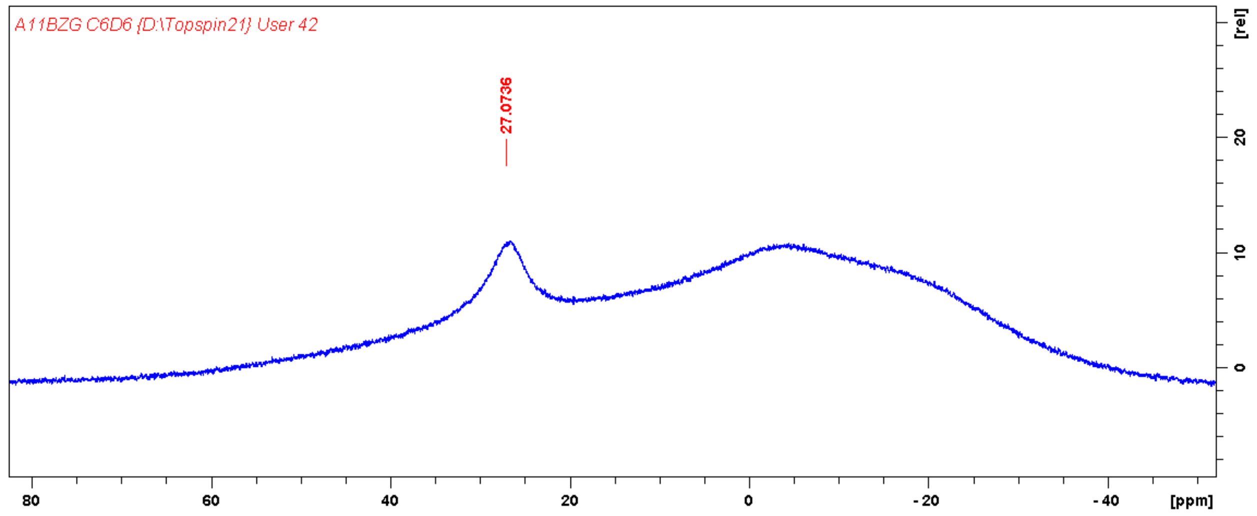

Figure S12. ^{11}B NMR (160 MHz, C_6D_6) spectrum of ^{Me}CAAC=NBBrDur (**4**)

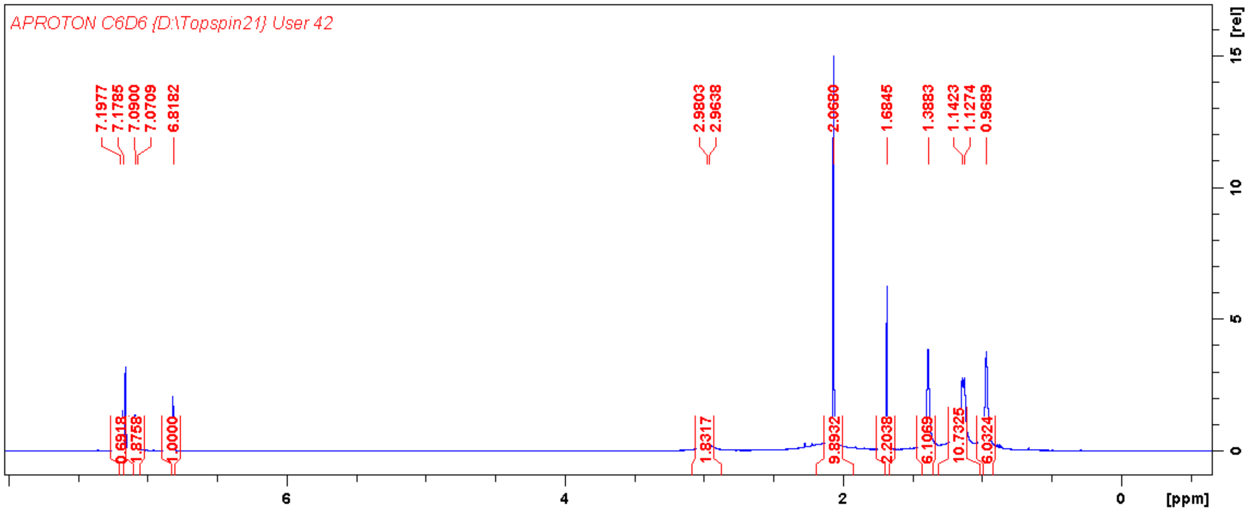

Figure S13. [11]H NMR (400 MHz, C_6D_6) spectrum of ^{Me}CAAC=NBBrDur (**4**)

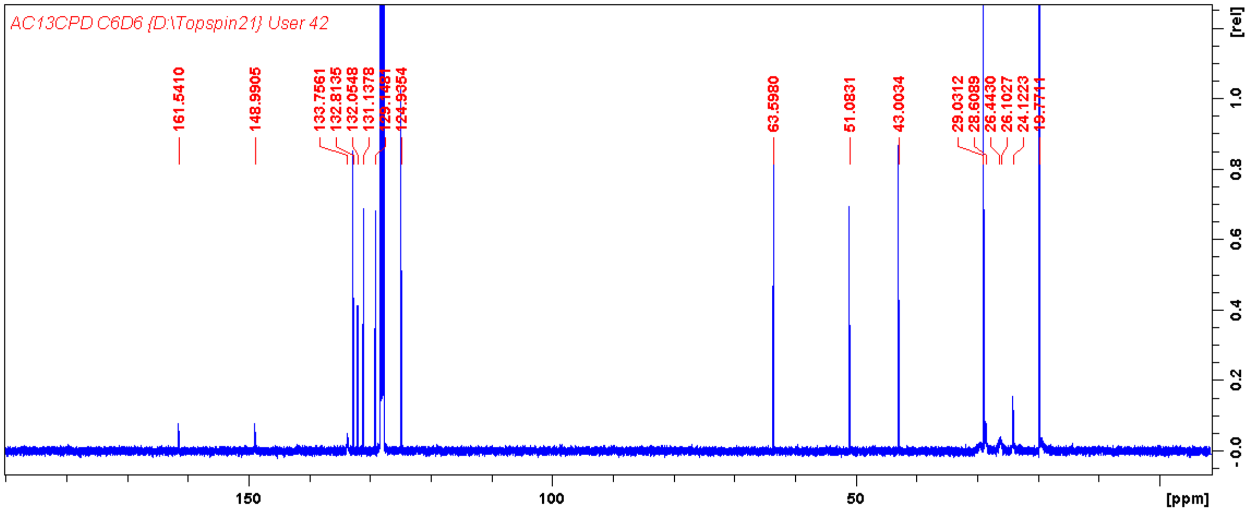

Figure S14. $^{13}C\{^1H\}$ NMR (100 MHz, C_6D_6) spectrum of ^{Me}CAAC=NBBrDur (**4**)

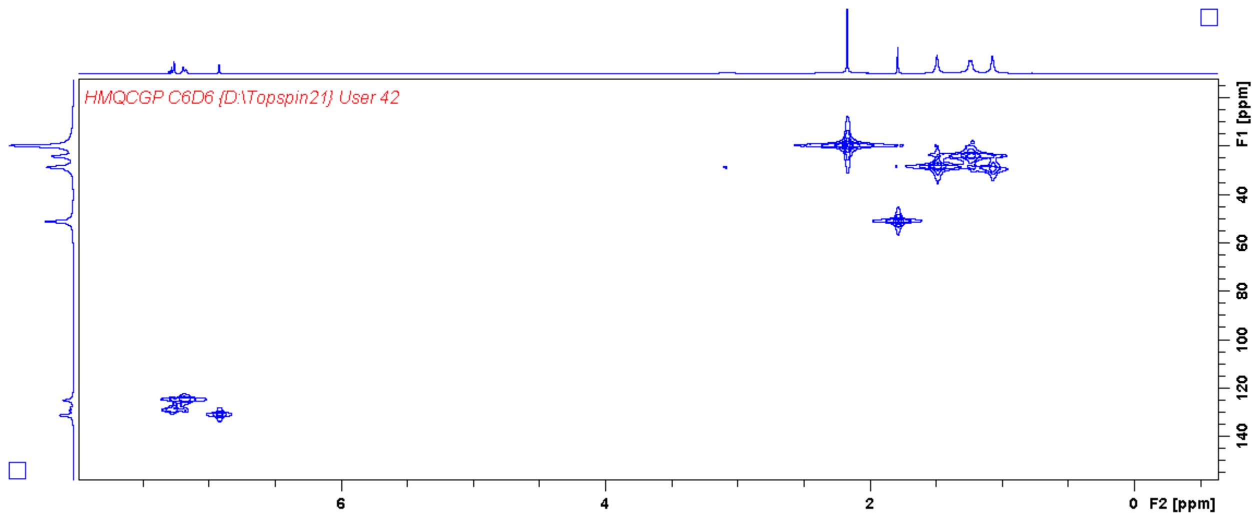

Figure S15. HMQC NMR (100 MHz, C_6D_6) spectrum of ^{Me}CAAC=NBBrDur (**4**)

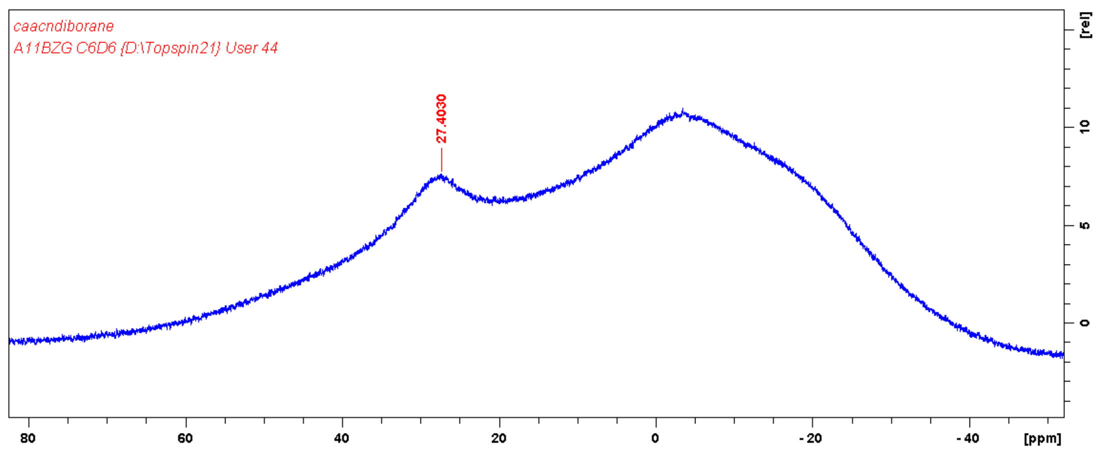

Figure S16. ^{11}B NMR (160 MHz, C_6D_6) spectrum of (^{Me}CAAC=N)$_2$Br$_2$Br$_2$ (**5**)

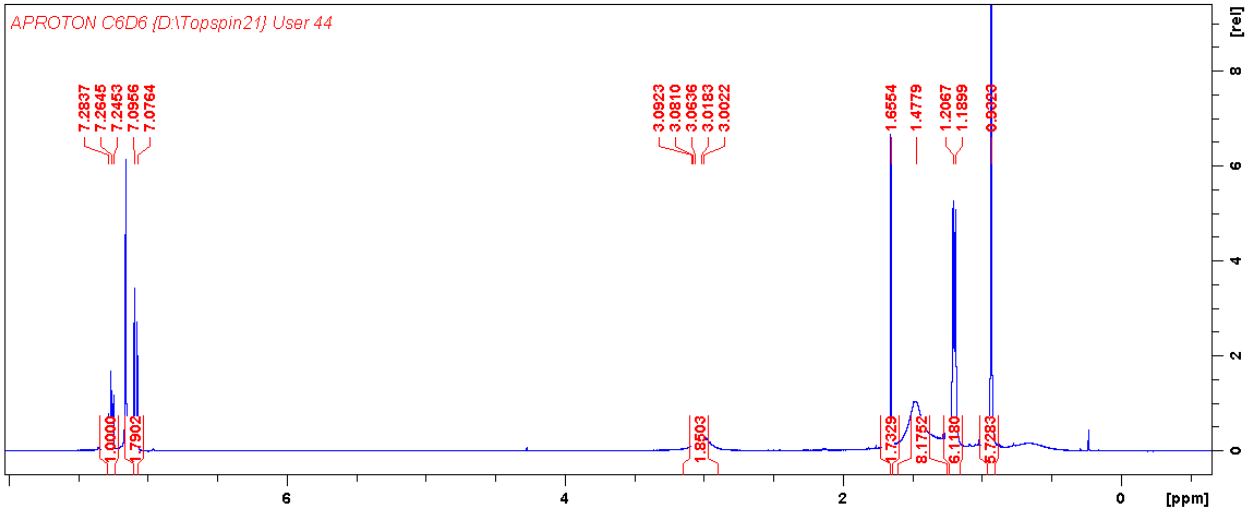

Figure S17. ^{11}H NMR (400 MHz, C_6D_6) spectrum of $(^{Me}CAAC{=}N)_2Br_2Br_2$ (**5**)

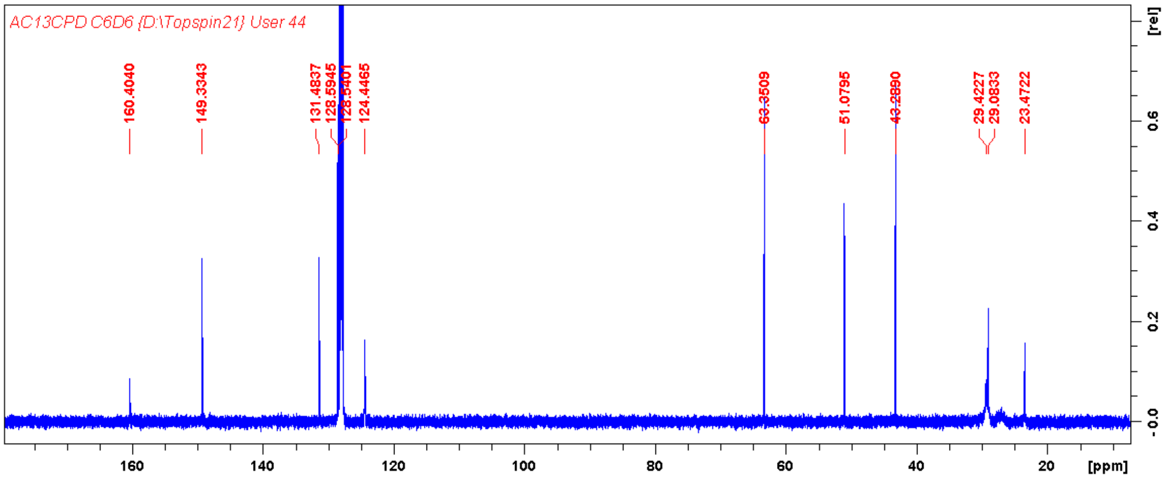

Figure S18. ^{13}C NMR (100 MHz, C_6D_6) spectrum of $(^{Me}CAAC{=}N)_2Br_2Br_2$ (**5**)

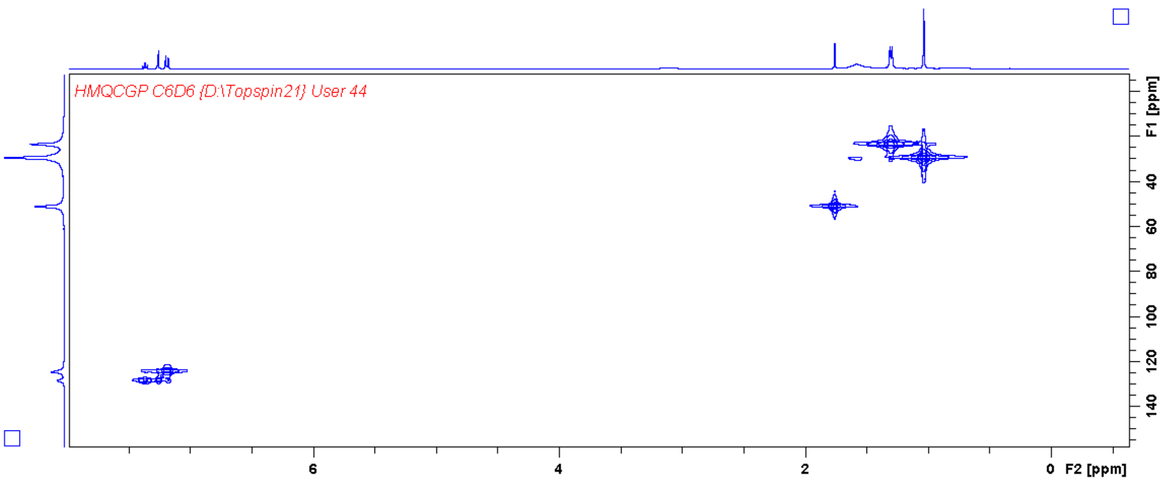

Figure S19. HMQC ^{13}C NMR (100 MHz, C_6D_6) spectrum of $(^{Me}CAAC{=}N)_2Br_2Br_2$ (**5**)

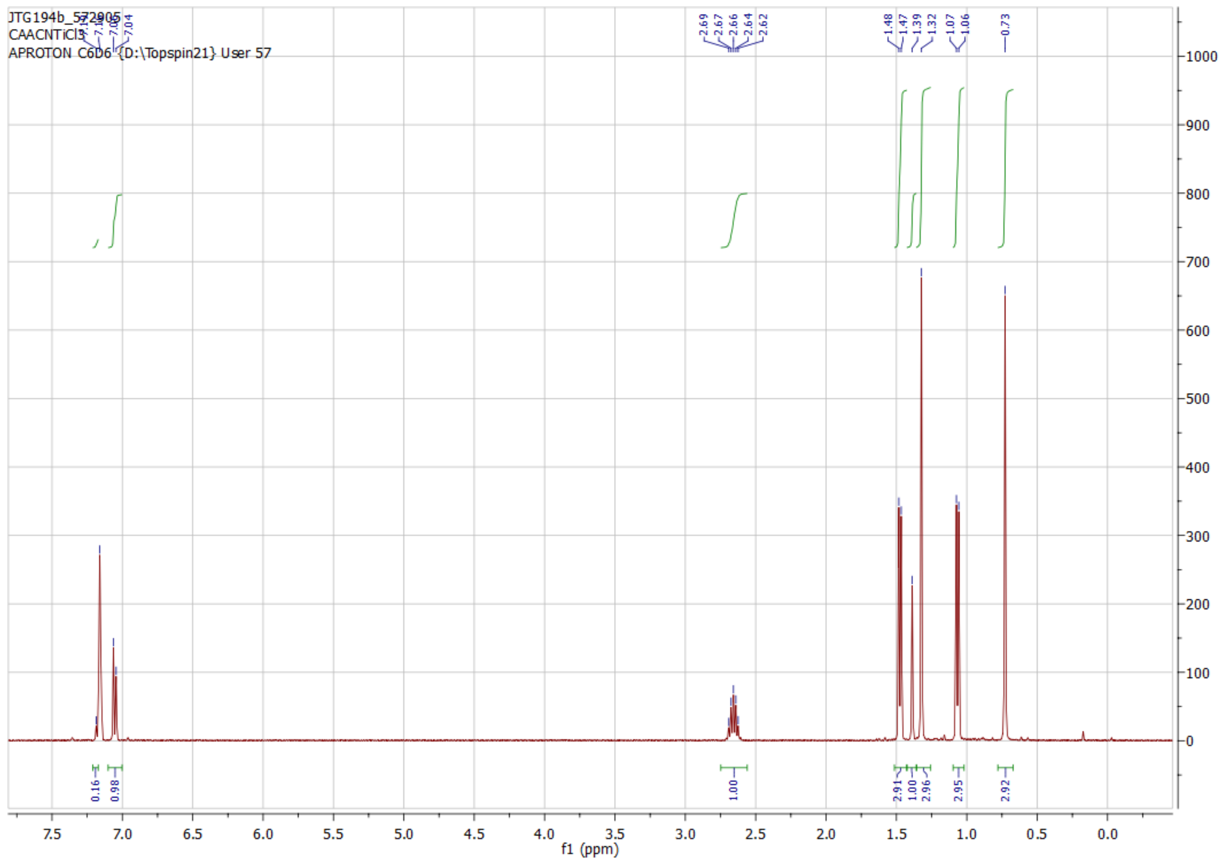

Figure S20. ^{1}H NMR (400 MHz, C_6D_6) spectrum of $^{Me}CAAC{=}NTiCl_3$ (**6**)

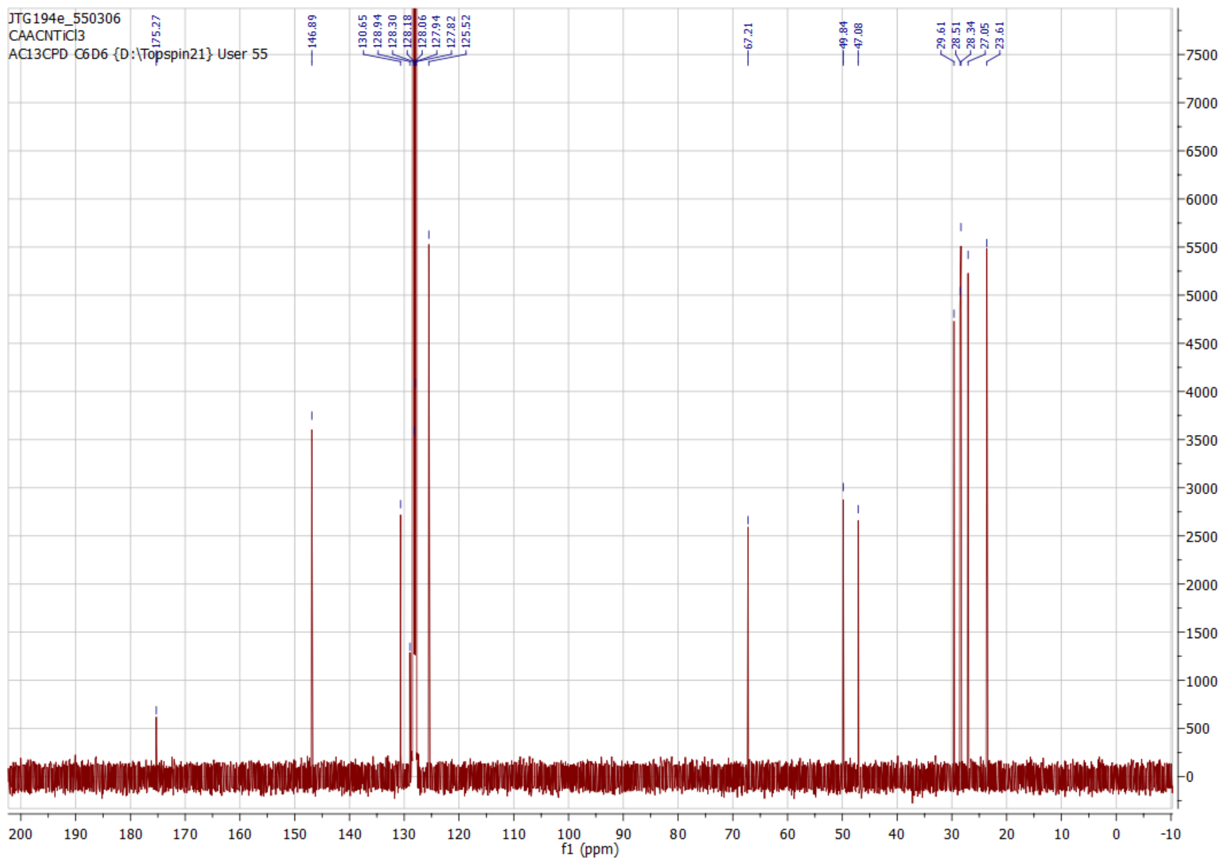

Figure S21. ^{13}C NMR (100 MHz, C_6D_6) spectrum of $^{Me}CAAC{=}NTiCl_3$ (**6**)

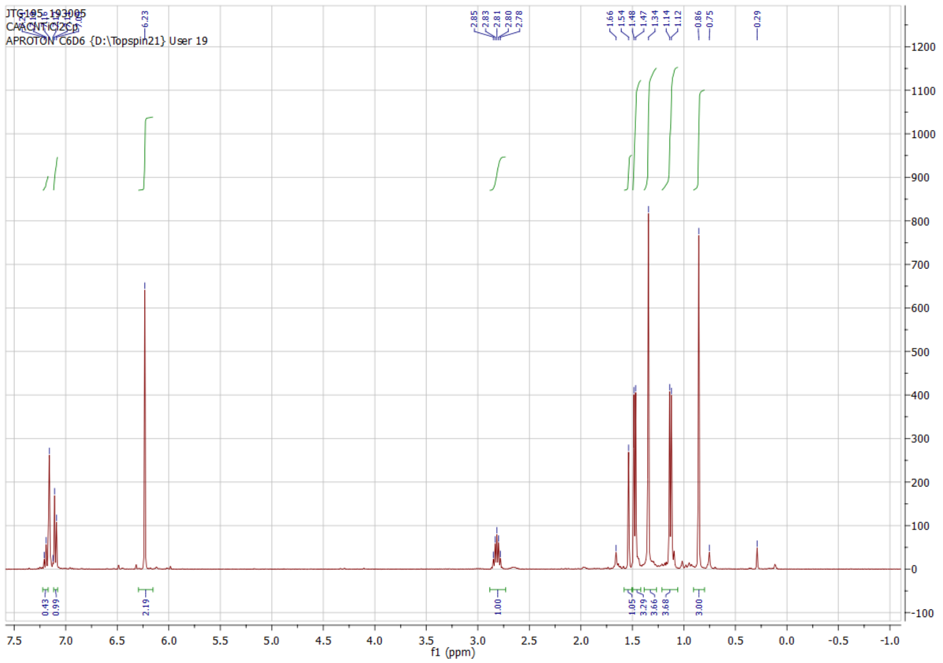

Figure S22. ^{1}H NMR (400 MHz, C_6D_6) spectrum of $^{Me}CAAC{=}NTiCl_2Cp$ (**7**)

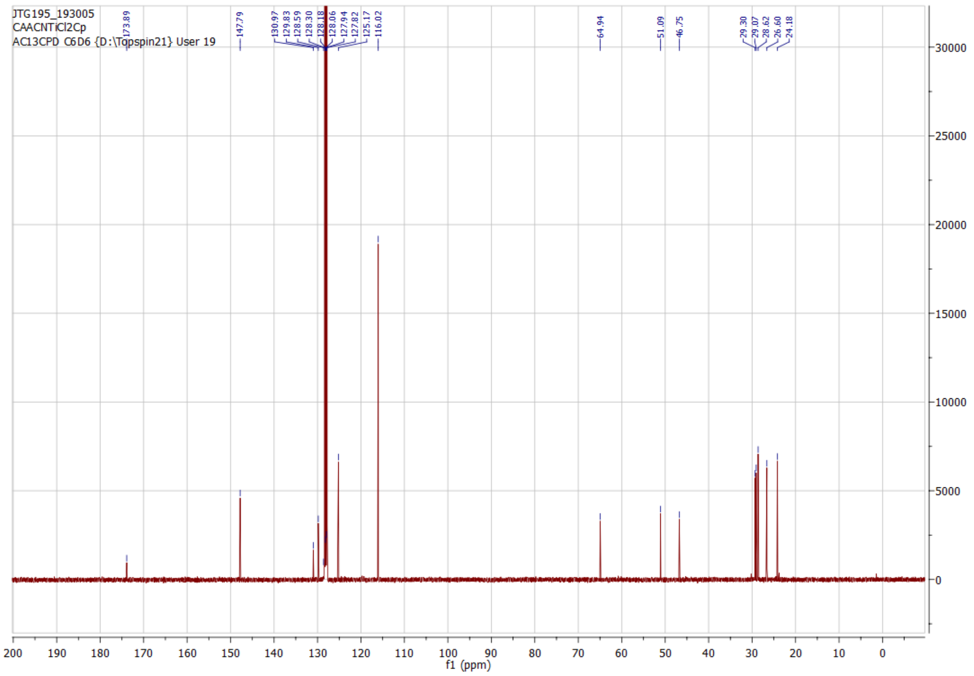

Figure S23. $^{13}C\{^{1}H\}$ NMR (100 MHz, C_6D_6) spectrum of $^{Me}CAAC{=}NTiCl_2Cp$ (**7**)

Mass Spectra

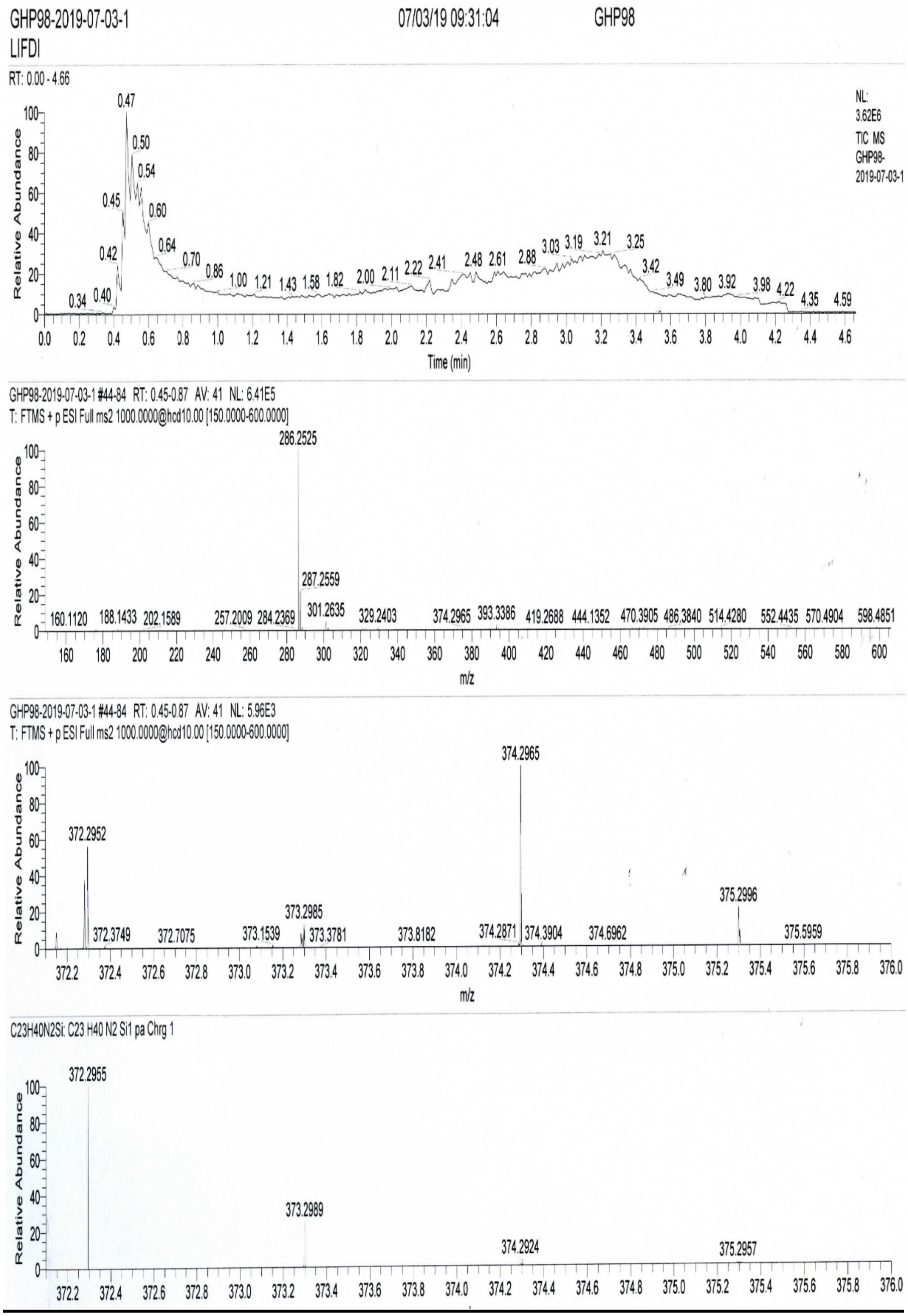

Figure S24. Mass spectrometry analysis of ^{Me}CAAC=NSiMe$_3$ (**1**)

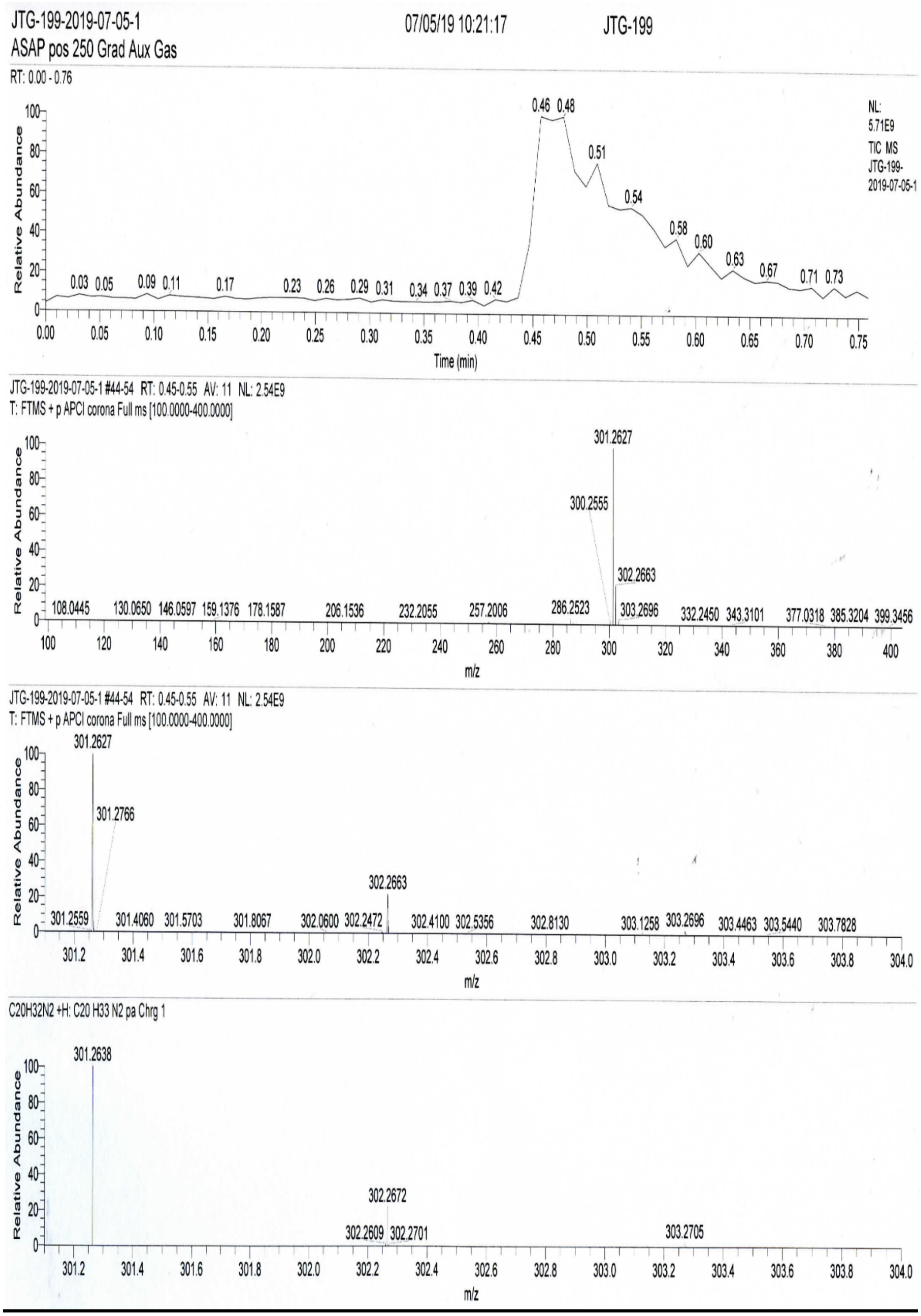

Figure S25. Mass spectrometry analysis of ^{Me}CAAC=NH (**2**)

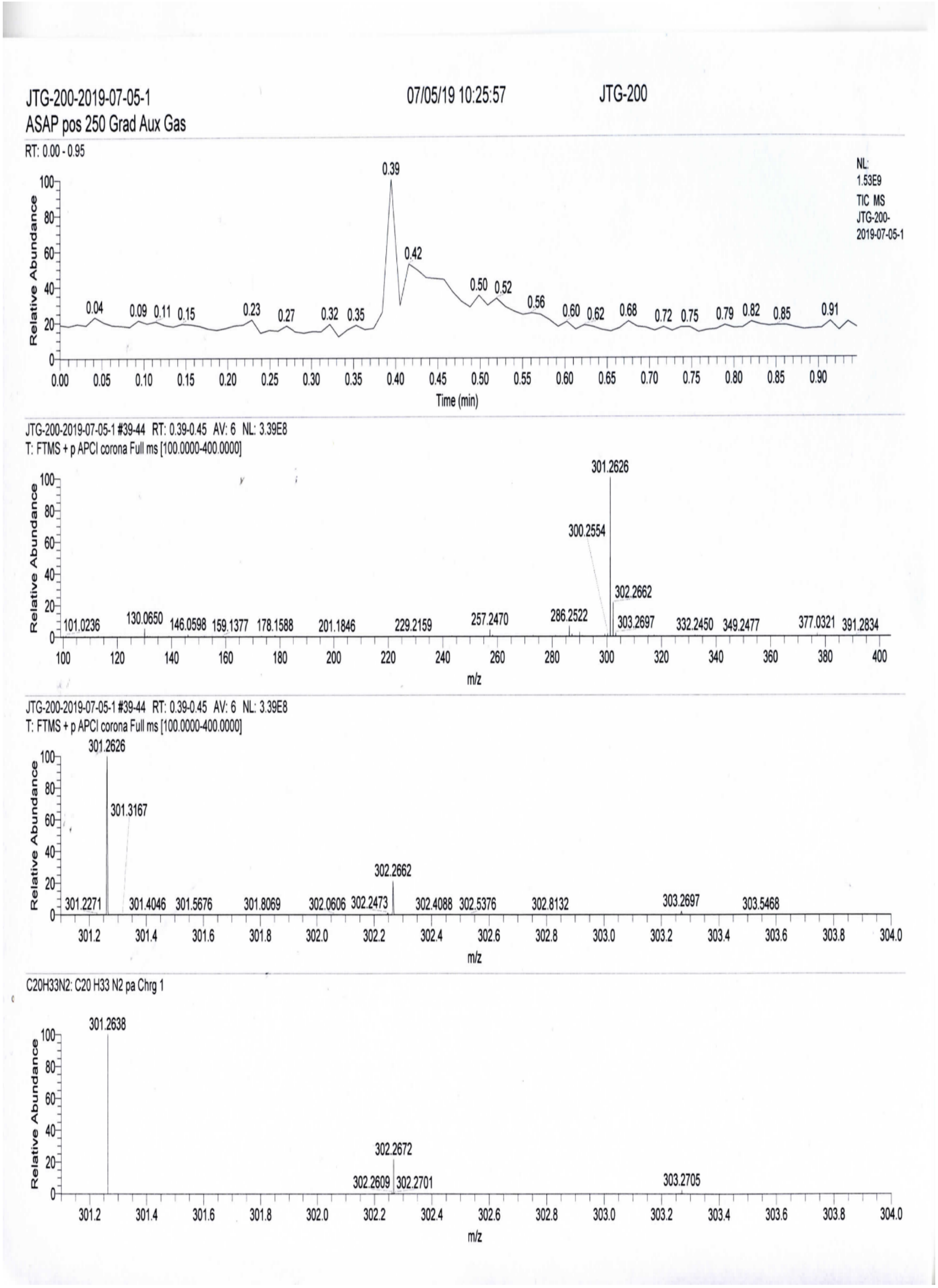

Figure S26. Mass spectrometry analysis of ($[^{Me}CAAC{=}NH_2]N_3)_2 \cdot H_2O$ (**3**)

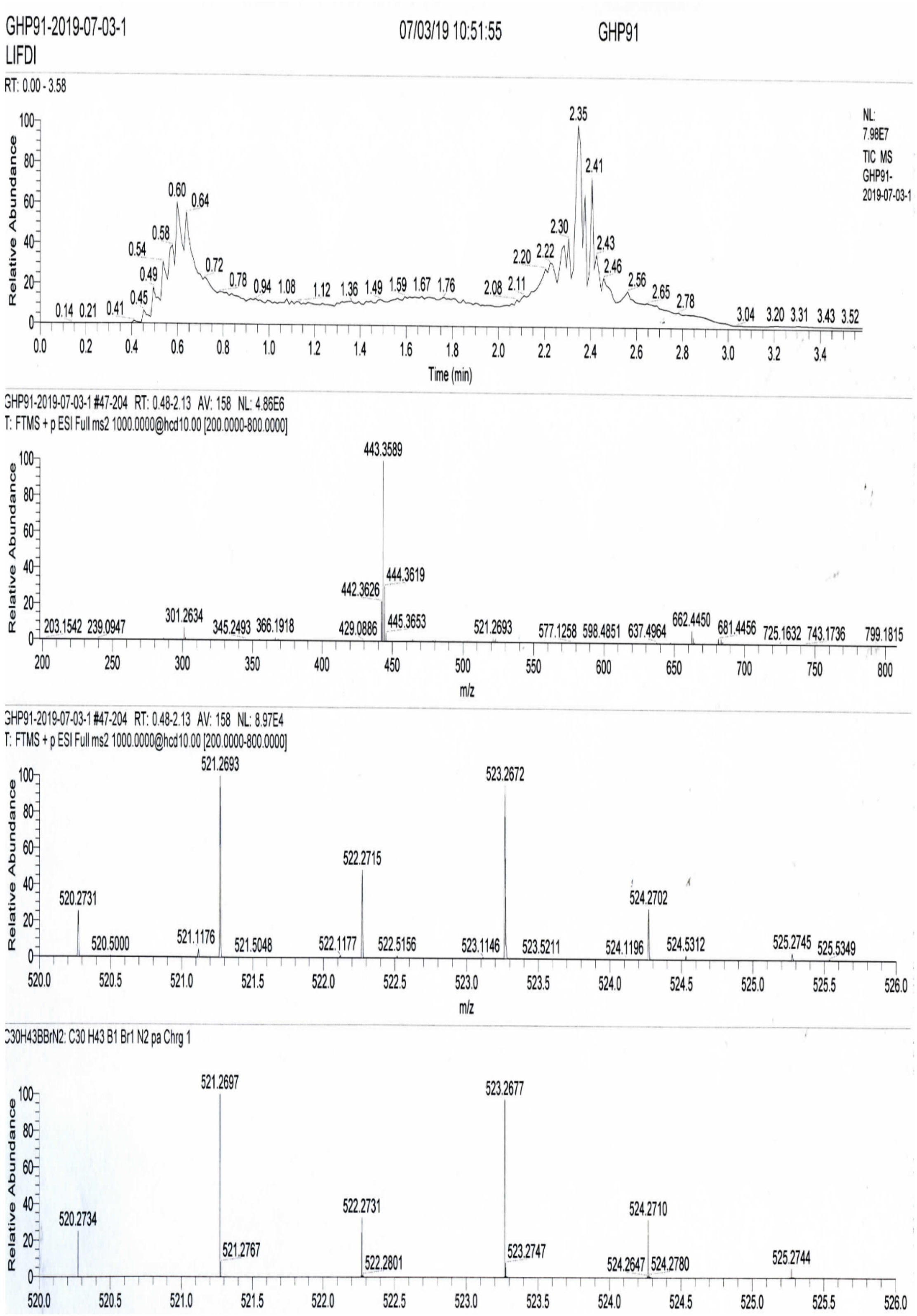

Figure S27. Mass spectrometry analysis of ^{Me}CAAC=NBBr(Dur) (**4**)

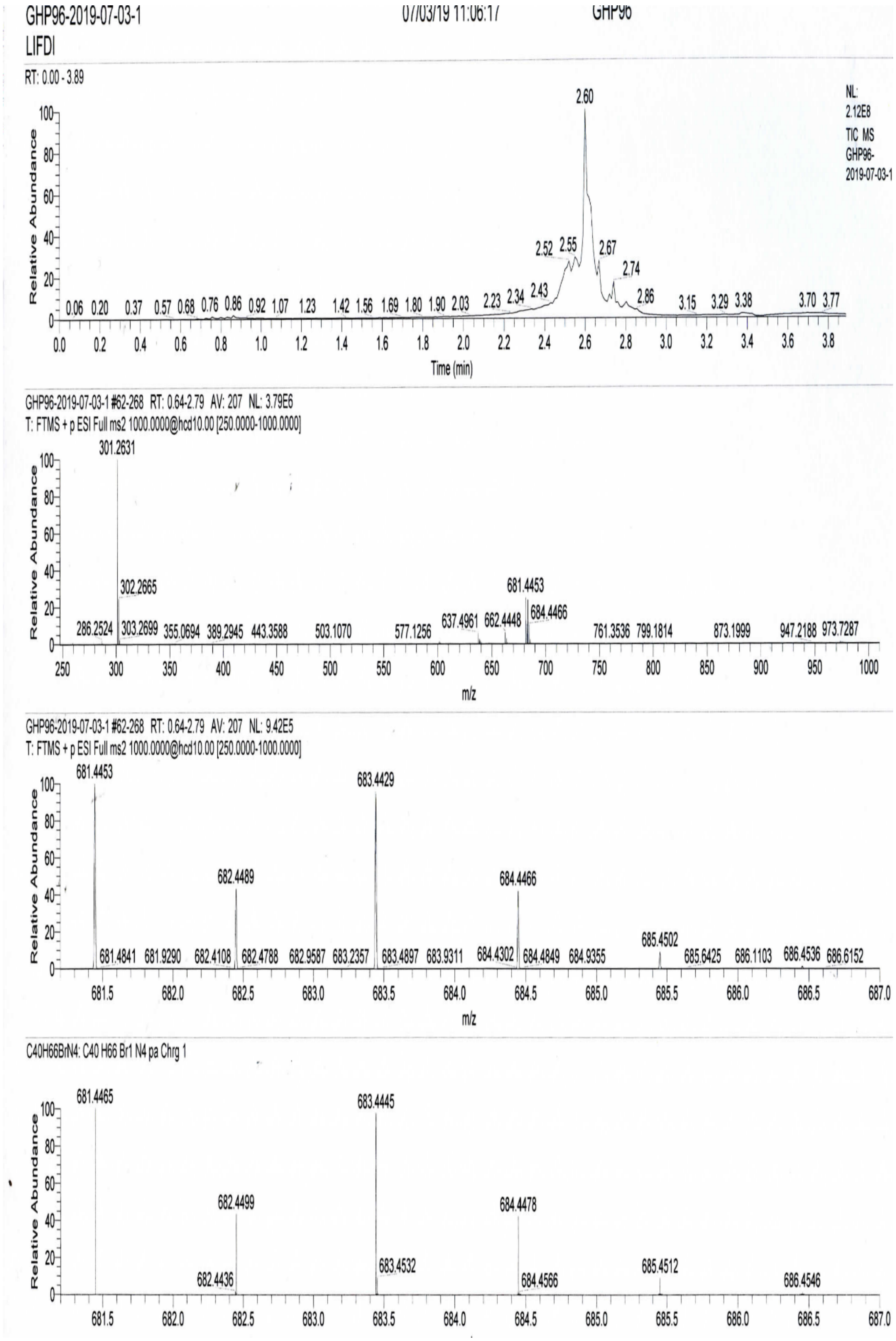

Figure S28. Mass spectrometry analysis of (^{Me}CAAC=N)$_2$B$_2$Br$_2$ (**5**)

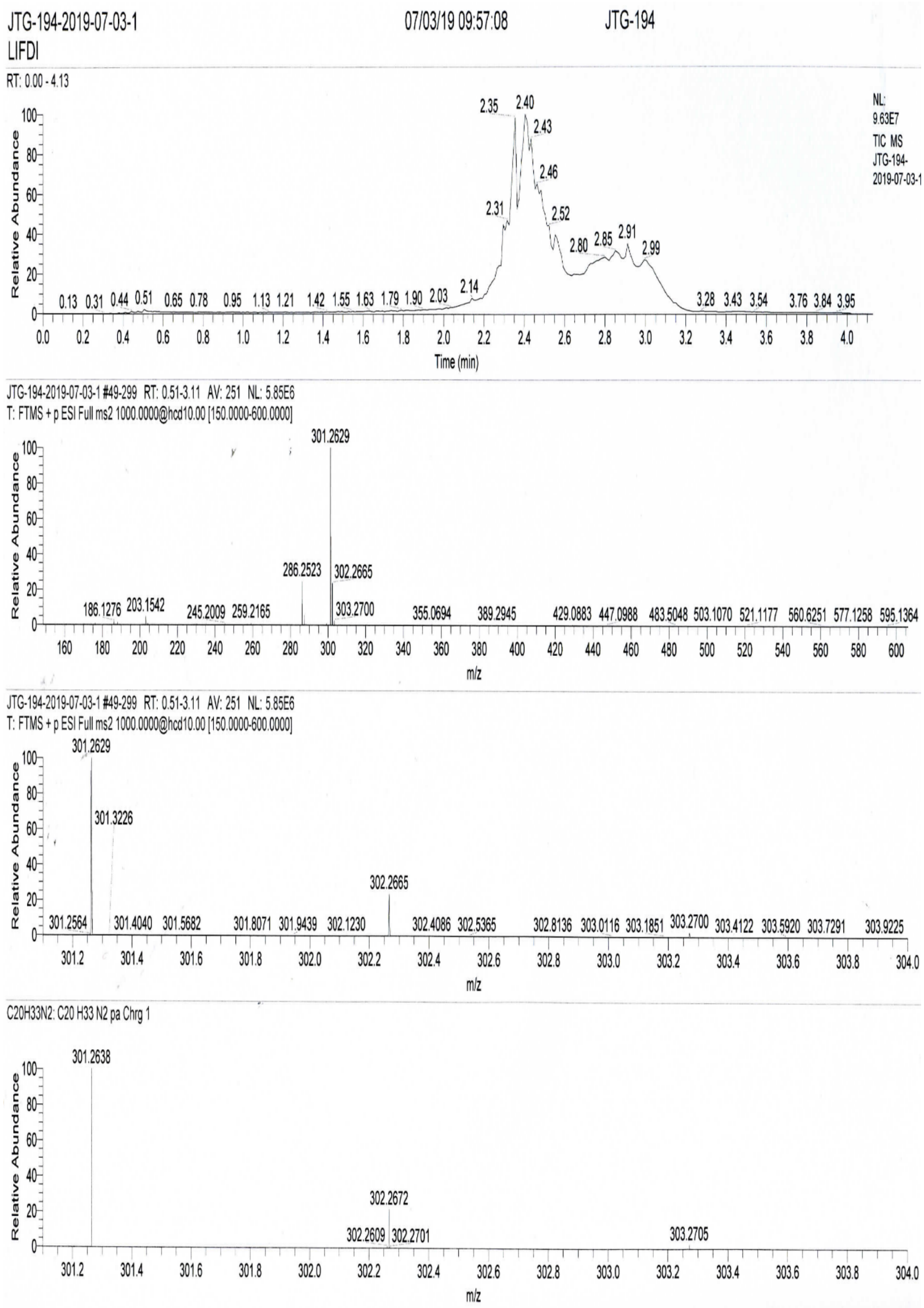

Figure S29. Mass spectrometry analysis of ^{Me}CAAC=NTiCl$_3$ (**6**)

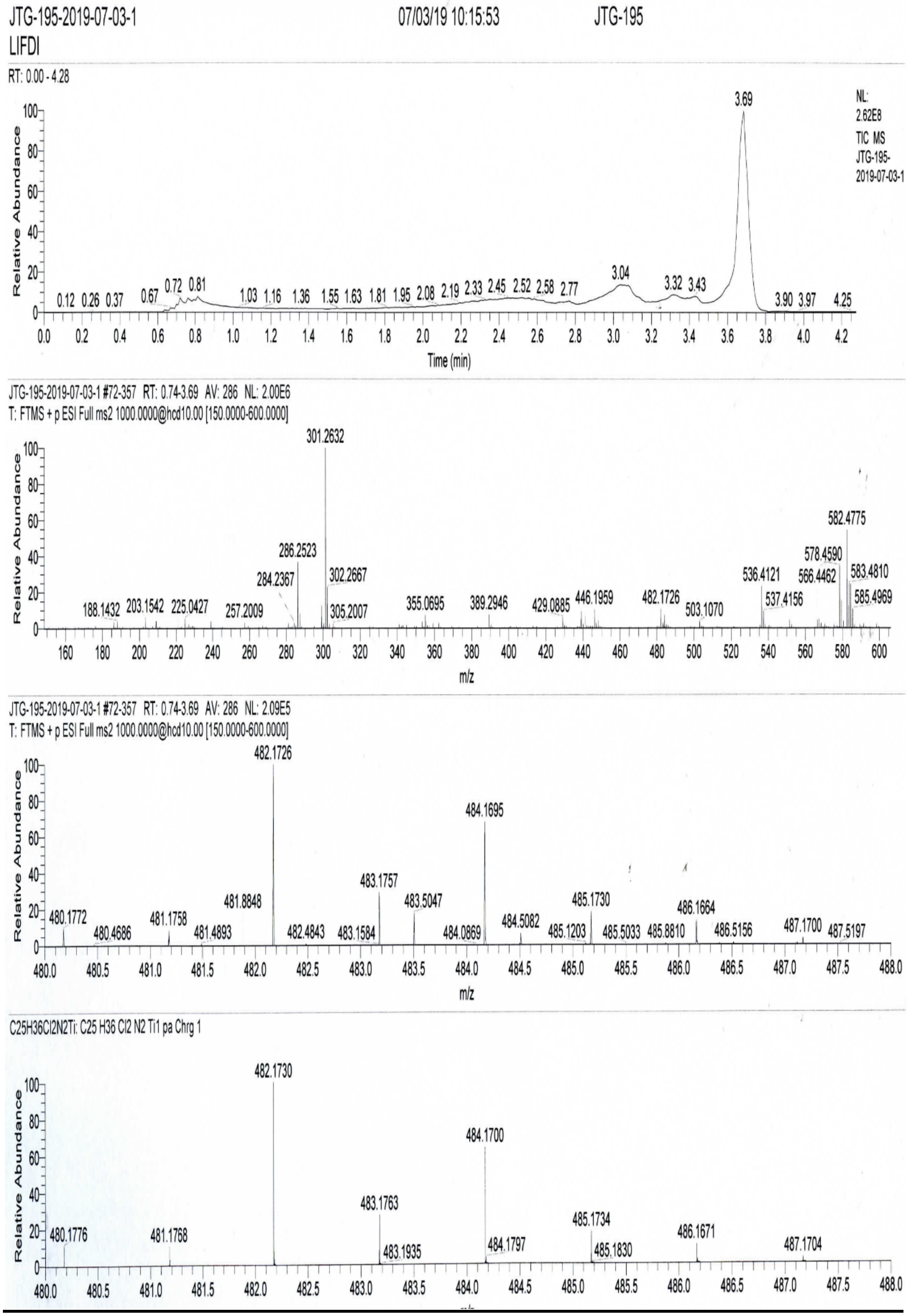

Figure S30. Mass spectrometry analysis of ^{Me}CAAC=NTiCl$_2$Cp (**7**)

Crystallographic Details

Crystal structure determination

The crystal data of ([^{Me}CAAC=NH$_2$]N$_3$)$_2$·H$_2$O (**3**) and ^{Me}CAAC=NTiCl$_3$ (**6**) were collected on a BRUKER SMART-APEX diffractometer with a CCD area detector and graphite monochromated Mo$_{K\alpha}$ radiation. The crystal data of ^{Me}CAAC=NH (**2**) were collected on a BRUKER X8-APEX II diffractometer with a CCD area detector and multi-layer mirror monochromated Mo$_{K\alpha}$ radiation. The crystal data of ^{Me}CAAC=NTiCpCl$_2$ (**7**) were collected on a BRUKER X8-APEX II diffractometer with a CCD area detector and graphite monochromated Mo$_{K\alpha}$ radiation. The crystal data of ^{Me}CAAC=NSiMe$_3$ (**1**), ^{Me}CAAC=NH·HOSiMe$_3$ (**2·HOSiMe$_3$**), ^{Me}CAAC=NBBrDur (**4**) and (^{Me}CAAC=N)$_2$(B$_2$Br$_2$) (**5**) were collected on a Bruker D8 Quest diffractometer with a CMOS area detector and multi-layer mirror monochromated Mo$_{K\alpha}$ radiation. The structures were solved using the intrinsic phasing method,[1] refined with the ShelXL program[2] and expanded using Fourier techniques. All non-hydrogen atoms were refined anisotropically. Hydrogen atoms were included in structure factor calculations. All hydrogen atoms were assigned to idealized geometric positions except where noted.

Crystal data for **^{Me}CAAC=NSiMe$_3$ (1)**: C$_{23}$H$_{40}$N$_2$Si, M_r = 372.66, colorless block, 0.323×0.296×0.201 mm^3, monoclinic space group $P2_1/c$, a = 14.2743(6) Å, b = 13.372(2) Å, c = 12.409(2) Å, β = 90.198(2)°, V = 2368.5(6) Å^3, Z = 4, ρ_{calcd} = 1.045 g·cm^{-3}, μ = 0.108 mm^{-1}, F(000) = 824, T = 100(2) K, R_1 = 0.0375, wR^2 = 0.0948, 11495 independent reflections [2θ≤72.67°] and 247 parameters. The structure was refined using TWIN keyword (matrix: TWIN -1 0 0 0 -1 0 0 0 1 2). The BASF parameter was refined to 19.166%. CCDC-1956002

Crystal data for **^{Me}CAAC=NHOHSiMe$_3$ (2·OHSiMe$_3$)**: C$_{23}$H$_{42}$N$_2$OSi, M_r = 390.67, colorless plate, 0.338×0.141×0.118 mm^3, triclinic space group $P\,\bar{1}$, a = 10.0060(10) Å, b = 10.9255(11) Å, c = 11.8835(11) Å, α = 96.680(4)°, β = 107.809(4)°, γ = 97.174(4)°, V = 1210.6(2) Å^3, Z = 2, ρ_{calcd} = 1.072 g·cm^{-3}, μ = 0.111 mm^{-1}, F(000) = 432, T = 100(2) K, R_1 = 0.0876, wR^2 = 0.1728, 4917 independent reflections [2θ≤53.044°] and 263 parameters. The atomic displacement parameters of all atoms were restraint with RIGU keyword in ShelXL input ('enhanced rigid bond' restraint for all bonds in the connectivity list. Standard values of 0.004 for both parameters s1 and s2 were used). CCDC-1956007

Crystal data for **^{Me}CAAC=NH (2)**: $C_{20}H_{32}N_2$, M_r = 300.47, colorless block, 0.198×0.161×0.054 mm^3, monoclinic space group $P2_1/n$, a = 9.269(3) Å, b = 16.817(6) Å, c = 11.982(3) Å, β = 95.971(15)°, V = 1857.6(10) Å^3, Z = 4, ρ_{calcd} = 1.074 g·cm^{-3}, μ = 0.062 mm^{-1}, F(000) = 664, T = 100(2) K, R_1 = 0.0752, wR^2 = 0.1126, 3793 independent reflections [2θ≤52.814°] and 211 parameters. CCDC-1956008

Crystal data for **([^{Me}CAAC=NH$_2$]N$_3$)$_2$·H$_2$O (3)**: $C_{40}H_{68}N_{10}O$, M_r = 705.04, colorless block, 0.391×0.159×0.097 mm^3, monoclinic space group $C2/c$, a = 13.436(6) Å, b = 12.448(6) Å, c = 24.605(13) Å, β = 98.97(3)°, V = 4065(3) Å^3, Z = 4, ρ_{calcd} = 1.152 g·cm^{-3}, μ = 0.072 mm^{-1}, F(000) = 1544, T = 100(2) K, R_1 = 0.0648, wR^2 = 0.1040, 4493 independent reflections [2θ≤54.258°] and 251 parameters. The hydrogen atoms bonded to N and O atoms were located in the difference map. CCDC-1956009

Crystal data for **^{Me}CAAC=NBBrDur (4)**: $C_{30}H_{44}BBrN_2$, M_r = 523.39, colorless Block, 0.304×0.262×0.244 mm^3, orthorhombic space group $Pbca$, a = 16.744(4) Å, b = 17.929(8) Å, c = 19.163(6) Å, V = 5753(3) Å^3, Z = 8, ρ_{calcd} = 1.209 g·cm^{-3}, μ = 1.449 mm^{-1}, F(000) = 2224, T = 106(2) K, R_1 = 0.0346, wR^2 = 0.0720, 7445 independent reflections [2θ≤57.432°] and 319 parameters. CCDC-1956003

Crystal data for **(^{Me}CAAC=N)$_2$(B$_2$Br$_2$) (5)**: $C_{46.42}H_{68.65}B_2Br_2N_4$, M_r = 864.18, colorless Block, 0.254×0.151×0.149 mm^3, monoclinic space group $P2_1/c$, a = 9.997(4) Å, b = 33.219(10) Å, c = 14.283(5) Å, β = 100.341(10)°, V = 4666(3) Å^3, Z = 4, ρ_{calcd} = 1.230 g·cm^{-3}, μ = 1.772 mm^{-1}, F(000) = 1821, T = 106(2) K, R_1 = 0.0511, wR^2 = 0.0744, 9540 independent reflections [2□≤52.766°] and 521 parameters. The distances between atoms C47 and H47 were kept during refinement at the value of 0.95 using DFIX restraint. CCDC-1956004

Crystal data for **^{Me}CAAC=NTiCl$_3$ (6)**: $C_{20}H_{31}Cl_3N_2Ti$, M_r = 453.72, orange plate, 0.376×0.304×0.132 mm^3, monoclinic space group $P2_1/n$, a = 9.4697(15) Å, b = 15.119(2) Å, c = 16.366(4) Å, β = 92.43(2)°, V = 2341.1(8) Å^3, Z = 4, ρ_{calcd} = 1.287 g·cm^{-3}, μ = 0.715 mm^{-1}, F(000) = 952, T = 100(2) K, R_1 = 0.0428, wR^2 = 0.0951, 6601 independent reflections [2θ≤59.354°] and 243 parameters. CCDC-1956005

Crystal data for ^{Me}**CAAC=NTiCl$_2$Cp (7)**: $C_{25}H_{36}Cl_2N_2Ti$, M_r = 483.36, orange Plate, 0.267×0.18×0.054 mm^3, monoclinic space group $P2_1/c$, a = 17.798(5) Å, b = 9.4680(15) Å, c = 15.061(4) Å, β = 95.201(18)°, V = 2527.4(11) Å^3, Z = 4, ρ_{calcd} = 1.270 g·cm^{-3}, μ = 0.564 mm^{-1}, F(000) = 1024, T = 100(2) K, R_1 = 0.0839, wR^2 = 0.1817, 5172 independent reflections [2$\theta \leq$52.866°] and 280 parameters. The structure was refined using TWIN keyword (matrix: TWIN 1 0 0.214 0 -1 0 0 0 -1 2). The BASF parameter was refined to 3.274%. CCDC-1956006

References

[1] ShelXT, G. Sheldrick, *Acta Cryst.* **2015**, *A71*, 3–8.

[2] G. Sheldrick, *Acta Cryst.* **2008**, *A64*, 112–122.
